# Caffeic acid *N*-[3,5-bis(trifluoromethyl)phenyl] amide as a non-steroidal inhibitor for steroid 5α-reductase type 1 using a human keratinocyte cell-based assay and molecular dynamics

**DOI:** 10.1038/s41598-022-25335-7

**Published:** 2022-12-02

**Authors:** Aye Chan Khine Lin, Ponsawan Netcharoensirisuk, Kamonpan Sanachai, Warongrit Sukma, Chaisak Chansriniyom, Chatchai Chaotham, Wanchai De-Eknamkul, Thanyada Rungrotmongkol, Supakarn Chamni

**Affiliations:** 1 Pharmaceutical Sciences and Technology Program, Faculty of Pharmaceutical Sciences, Chulalongkorn University, Bangkok, 10330 Thailand; 2 Department of Pharmacognosy and Pharmaceutical Botany, Faculty of Pharmaceutical Sciences, Chulalongkorn University, Bangkok, 10330 Thailand; 3 Natural Products and Nanoparticles Research Unit (NP2), Chulalongkorn University, Bangkok, 10330 Thailand; 4 Natural Product Biotechnology Research Unit, Chulalongkorn University, Bangkok, 10330 Thailand; 5 Department of Biochemistry, Faculty of Science, Khon Kaen University, Khon Kaen, 40002 Thailand; 6 Department of Biochemistry and Microbiology, Faculty of Pharmaceutical Sciences, Chulalongkorn University, Bangkok, 10330 Thailand; 7 Center of Excellence in Cancer Cell and Molecular Biology, Faculty of Pharmaceutical Sciences, Chulalongkorn University, Bangkok, 10330 Thailand; 8 Center of Excellence in Structural and Computational Biology, Department of Biochemistry, Faculty of Science, Chulalongkorn University, Bangkok, 10330 Thailand; 9 Program in Bioinformatics and Computational Biology, Graduate School, Chulalongkorn University, Bangkok, 10330 Thailand

**Keywords:** Drug discovery, Medicinal chemistry

## Abstract

Caffeic acid derivatives containing amide moieties similar to those of finasteride and dutasteride were synthesized. An in vitro inhibitory activity evaluation of caffeic acid (**1**) and its amide derivatives (**2** − **4**) against the steroid 5α-reductase type 1 (SRD5A1) produced by human keratinocyte cells coupled with the non-radioactive high-performance thin-layer chromatography detection revealed that caffeic acid *N*-[3,5-bis(trifluoromethyl)phenyl] amide (**4**) was a promising non-steroidal suppressor, with a half-maximal inhibitory concentration (IC_50_) of 1.44 ± 0.13 µM and relatively low cytotoxicity with an IC_50_ of 29.99 ± 8.69 µM. The regulatory role of compound **4** against SRD5A1 involved both suppression of SRD5A1 expression and mixed mode SRD5A1 inhibition. The K_i_ value of compound **4** was 2.382 µM based on the whole-cell kinetic studies under specific conditions. Molecular docking and molecular dynamics simulations with AlphaFold generated the human SRD5A1 structure and confirmed the stability of compound **4** at the SRD5A1 catalytic site with greater interactions, including hydrogen bonding of the key M119 amino-acid residue than those of finasteride and dutasteride. Thus, compound **4** shows the potential for further development as an SRD5A1 suppressor for androgenic alopecia treatment.

## Introduction

Steroid 5α-reductases, also known as 3-oxo-5α-steroid 4-dehydrogenases, are microsomal and nicotinamide adenine dinucleotide phosphate (NADPH)-dependent membrane-bound enzymes in the oxidoreductase family; they are involved in steroid metabolism. Currently, these enzymes are classified into types 1, 2, and 3^1^. The isozymes are produced in various human tissues and have diverse functional roles in human health and diseases. Steroid 5α-reductase type 1 (SRD5A1) is mostly found in hair follicles, scalp, sebaceous glands, non-genital skin, and liver; type 2 (SRD5A2) is abundant in genital tissues and scalp^2^; and type 3 (SRD5A3) is detected in benign and malignant tissues^3^. Both SRD5A1 and SRD5A2 exhibit amino-acid sequence similarity ranging from 40 to 60%^4^. They significantly control the conversion of testosterone to dihydrotestosterone (DHT), a more potent androgen. Overexpression of the steroid 5α-reductase leads to DHT overproduction, causing androgen-dependent diseases, including benign prostatic hyperplasia, hirsutism, androgenic alopecia, and prostate cancer^1^. Both male and female patients with androgenic alopecia have high levels of SRD5A1 and SRD5A2 in frontal and occipital hair follicles. SRD5A1 was found as the major isozyme in scalp-hair follicles, in which SRD5A1 is approximately five times higher than SRD5A2^5^. Therefore, SRD5A1 serves as a crucial target for the treatment of androgenic alopecia by using the orally administered drugs, finasteride and dutasteride^6–10^. However, basic research and clinical studies reported several adverse effects of the steroidal inhibitors, finasteride and dutasteride, including impotence, abnormal ejaculation, abnormal sexual function, gynecomastia, and severe myopathy^6^. Therefore, there is a need to identify other novel steroid 5α-reductase inhibitors. Steroidal and non-steroidal inhibitors have been continuously reported in the past few decades^11–16^. Notably, a group of non-steroidal inhibitors are highly selective and exhibit lower interactions with other enzymes and receptors than steroid 5α-reductases in the steroidal endocrine system.

Some natural compounds isolated from medicinal plants are promising inhibitors of steroid 5α-reductase with few side effects. These compounds include avicequinone C, a furanonaphthoquinone from *Avicennia marina*^17^; osthenol, a coumarin from *Angelica koreana*^18^; physcion, a dihydroxyanthraquinone from *Polygonum multiflorum*^19^; and piperine, an alkaloid from *Piper nigrum*^20^. Moreover, polyphenols, such as curcumin from *Curcuma longa*^21^ and theaflavin-3,3′-digallate from *Camellia sinensis* (green tea)^22^, and triterpenoids, such as soyasaponin I and kaikasaponin III from *Puerariae thomsonii*^23^, exhibit moderate steroid 5α-reductase inhibition.

Building on previous studies, a method to determine a compound’s inhibitory effect on SRD5A1 using a cell-based assay with human-hair dermal papilla cells (HHDPCs)^24,25^ or human keratinocytes (HaCaTs)^17^ using non-radioactive and direct DHT detection by high-performance thin-layer chromatography (HPTLC) was developed. A HaCaT cell-based assay showed that avicequinone C, a natural furanonaphthoquinone isolated from *Avicennia marina,* was discovered as a promising SRD5A1 inhibitor with a half-maximal inhibitory concentration (IC_50_) of 4.45 ± 0.42 µM^17^.

Caffeic acid, also known as 3,4-dihydroxycinnamic acid, belongs to a natural class of hydroxycinnamic acids found in several medicinal plants, fruits, and vegetables. Interestingly, caffeic acid and its derivatives exhibit many biological activities, especially antioxidant and anti-inflammatory activities^26–30^. Moreover, the three-dimensional (3D) pharmacophore model of caffeic acid amides displays unique hydrophobic- and hydrophilic-binding interactions regarding the 3,4-dihydroxycinnamoyl scaffold against nitric oxide (NO) targets^31^. Recently, caffeic acid is reported as an active substance in ethanol-extracted propolis that significantly stimulated keratinocyte proliferation and hair growth in mice via anagen induction and hair elongation^32^. Thus, the 3,4-dihydroxycinnamic motif could be studied for its structure–activity relationship against SRD5A1 inhibition to identify candidates for treatment of androgenic alopecia.

To date, the crystal structure of human SRD5A1 is unknown^33^. The lack of a crystal structure of the human SRD5A1 adversely affects our ability to understand the mechanism of the enzyme’s activity and delays the discovery and development of effective inhibitors. Study of homologous protein structures of both SRD5A1 and SRD5A2 revealed a conserved catalytic site in steroid 5α-reductases catalyzing NADPH-mediated steroids reduction involving their signature Q, E, and Y residues^34^. The crystal structure of human SRD5A2 in complex with finasteride and NADP (PDB: 7BW1) led to the discovery of the ligand-binding catalytic pocket^35^, in which the amino-acid residues E57 and Y91 are crucial hydrogen-bonding sites for reducing testosterone to DHT. Moreover, residue R114 forms a hydrogen bond with the *tert*-butylacetamide tail group of finasteride^35^. The findings of an in-silico study support the importance of a specific amide moiety that could selectively bind to catalytic pocket of steroid 5α-reductase.

**Figure 1:**
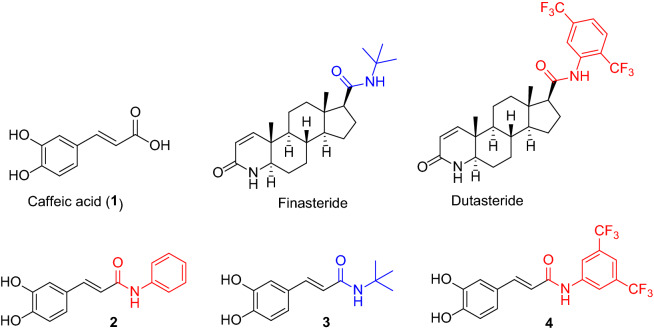
Structure of caffeic acid (**1**), its amide derivatives (**2** − **4**) and steroid 5α-reductase inhibited drugs, finasteride and dutasteride.

In this study, a series of caffeic acid derivatives were structurally designed, focusing on the amide structures similar to those in finasteride and dutasteride, including phenyl, *tert*-butyl, and trifluoromethyl substituted phenyl amide (Fig. 1). Caffeic acid was employed as the precursor in the synthesis of its amide derivatives by amidation. Next, the synthesized derivatives were evaluated for their potential as steroid 5α-reductase inhibitors using an anti-proliferative assay with the tetrazolium dye, 3-(4,5-dimethylthiazol-2-yl)-2,5-diphenyltetrazolium bromide **(**MTT), and the SRD5A1 inhibitory activity assay with HaCaTs and non-radioactive HPTLC^17^. The underlying molecular mechanism was examined via mRNA and protein expression. The kinetic inhibitory profiles were also investigated by employing whole-cell-based studies. Furthermore, the molecular docking and molecular dynamics AlphaFold-generated SRD5A1 structure predicted the possible binding sites and stability of the potent caffeic acid amides. These findings could lead to the discovery of a non-steroidal inhibitor for SRD5A1 for the use as a tropical treatment for androgenic alopecia.

## Results and discussion

### Synthesis of caffeic acid amide derivatives

**Figure 2:**
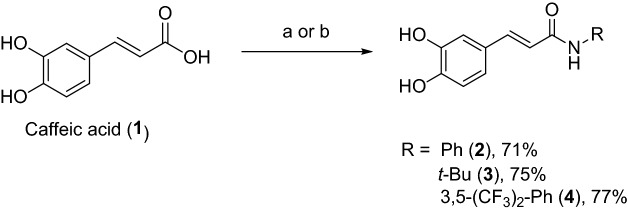
Syntheses of caffeic acid amide derivatives; (**a**) aniline or *tert*-butyl amine, EDCI^.^HCl, HOBt, THF, r.t., 16 h.; (**b**) *N*-[3,5-bis(trifluoromethyl)aniline], DCC, THF, reflux, 3 h.

The amide derivatives of caffeic acid with phenyl, *tert*-butyl, and *N*-[3,5-bis(trifluoromethyl) phenyl] substituents corresponding to compounds **2**, **3**, and **4**, respectively, were designed based on their distinct chemical structures. The phenyl group is an inductively withdrawing group, the *tert*-butyl is a bulky electron-donating group, and the *N*-[3,5-bis(trifluoromethyl)phenyl] is a sterically electron-withdrawing group. Caffeic acid (**1**) was employed as the precursor to react with amine reagents in the presence of amide coupling reagents under suitable conditions to generate the designed caffeic acid amide derivatives, compounds **2**, **3**, and **4,** with high yield (71%–77%) (Fig. 2).

### Identification of steroid 5α-reductases expressed in HaCaTs

**Figure 3:**
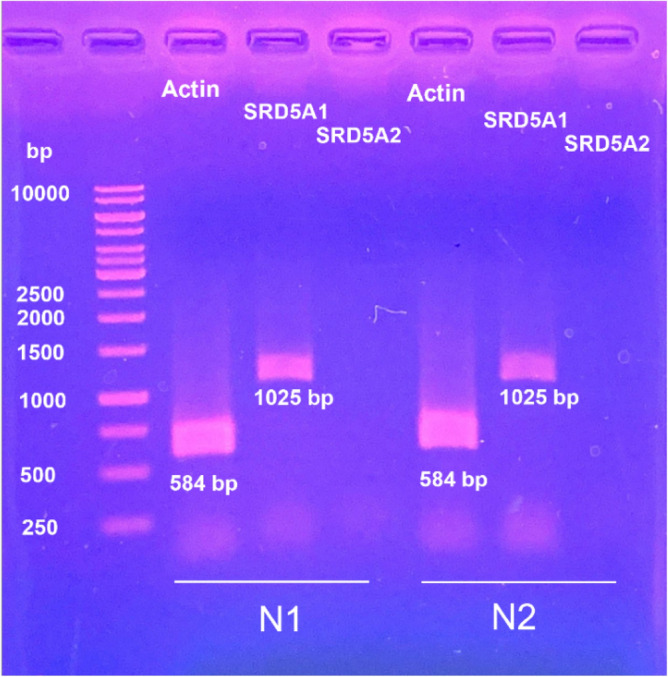
The expression of steroid 5α-reductase in human keratinocytes. RT-PCR analysis confirmed the presence of SRD5A1 and the absent of SRD5A2 in HaCaTs. N1, HaCaT cells passage 20 and N2, HaCaT cells passage number 26.

To identify the type of steroid 5α-reductase expressed in HaCaTs, mRNA levels of SRD5A1 and SRD5A2 were evaluated by reverse transcription polymerase chain reaction (RT-PCR) analysis. Figure 3 clearly shows that HaCaT cells abundantly expressed SRD5A1 and that the mRNA level of SRD5A2 was undetectable.

### Cytotoxicity of caffeic acid derivatives against human keratinocyte and dermal papilla cells

**Figure 4:**
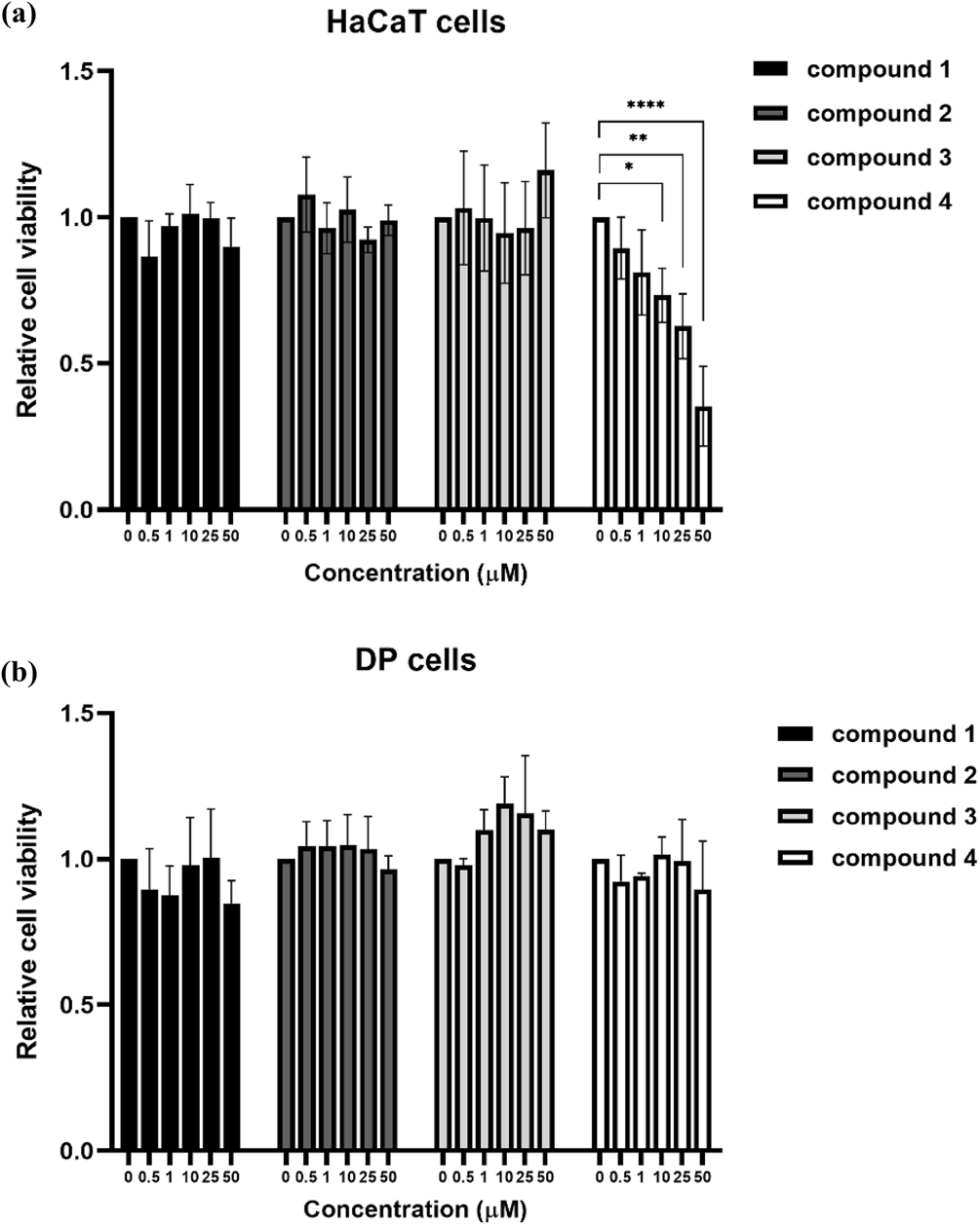
Cytotoxicity of caffeic acid (**1**) and its amide derivatives (compounds **2− 4)** against (**a**) HaCaT and (**b**) DP cells. The *N*-[3,5-bis(trifluoromethyl)phenyl]-containing amide **4** showed cytotoxicity at IC_50_ of 29.99 ± 8.69 µM against HaCaTs. The data of three independent experiments were represented as means ± standard deviation (SD). The viability of the compound-treated cells was compared to that of the untreated controls; *p < 0.05, **p < 0.001, and ****p < 0.0001.

Caffeic acid (**1**) and its amide analogs (**2 − 4**) were tested for their in vitro cytotoxicity to human keratinocytes (HaCaTs) and dermal papilla (DP) cells to identify their respective subtoxic concentrations. Caffeic acid (**1**) and its amide analogs containing phenyl (**2**) and *tert*-butyl (**3**) substituents exhibited no toxicity against HaCaT or DP cells at 0.5–50 µM (Fig. 4). However, the *N*-[3,5-bis(trifluoromethyl)phenyl]-containing analog (**4)** exhibited cytotoxicity against HaCaTs at IC_50_ 29.99 ± 8.69 µM and no toxicity to DP cells at 0.5–50 µM.

### In vitro evaluation of SRD5A1 inhibitory activity using HaCaT cell-based assay with HPTLC

Next, compounds **1**− **4** were screened for their potential inhibition of SRD5A1 activity in a HaCaT cell-based assay with direct DHT detection by HPTLC. This was evaluated at the sub-cytotoxic concentrations of compound **1**− **3** at 20 µM and amide **4** at 1 µM. Interestingly, the *N*-[3,5-bis(trifluoromethyl)phenyl]-containing amide (**4**), with a similar amide moiety to dutasteride, significantly inhibited the production of DHT by up to 46% at 1 µM, with high cell viability (88%). In contrast, caffeic acid (**1**) at 20 µM displayed a 30% inhibition of DHT production, with 86% cell viability, indicating a weaker inhibitory activity than compound **4**. In addition, the phenyl-containing amide **2** and the *tert*-butyl-containing amide **3** showed essentially no inhibition of the DHT production (Table 1). Testosterone and dutasteride, a substrate and an inhibitor of steroid 5α-reductase, respectively, were employed as experimental controls.

**Table 1:** Preliminary screening of SRD5A1 inhibitory activity of caffeic acid and its amide derivatives using the HaCaT cell-based assay with HPTLC.

Entry	Compound^a^	Concentration (µM)	% Cell viability ± SD	% Inhibition of DHT production ± SD
1	Testosterone	10	100	0
2	**1**	20	86.38 ± 5.70	30.02 ± 7.74
3	**2**	20	102.86 ± 0.40	4.84 ± 2.12
4	**3**	20	101.99 ± 2.09	2.00 ± 2.12
5	**4**	1.0	87.85 ± 1.13	45.67 ± 3.57
6	Dutasteride	2.5	88.14 ± 0.11	100

^a^Testosterone is an enzyme substrate. Compound **1** is caffeic acid. Compounds **2−4** are caffeic acid amide derivatives. Dutasteride is a steroid 5α-reductase inhibitor, which was used as a positive control.

**Figure 5:**
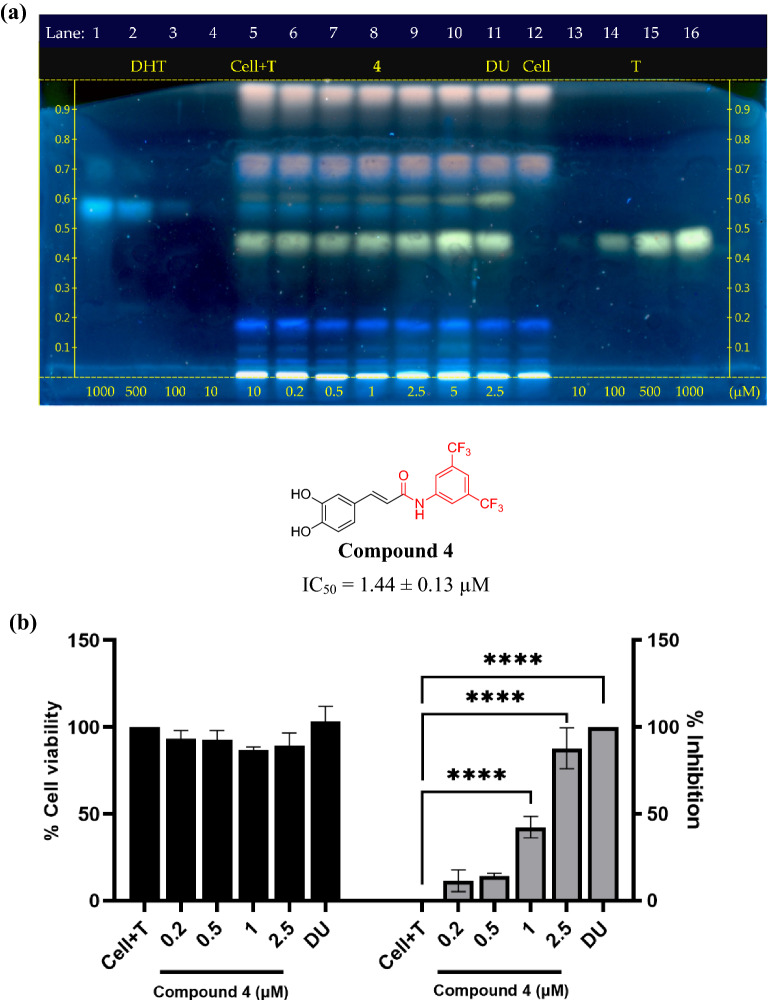
In vitro evaluation of the inhibition of SRD5A1 activity by the caffeic acid *N*-[3,5-bis(trifluoromethyl)phenyl] amide (**4**) using a HaCaT cell-based assay with direct DHT detection by HPTLC. (**a**) HPTLC chromatogram. Lanes 1−4, dihydrotestosterone (DHT) standards at different concentrations. Lane 5, HaCaTs (Cell) treated with 10 μM testosterone (T). Lanes 6 − 10, HaCaTs treated with compound **4** at different concentrations. Lane 11, HaCaTs treated with 2.5 μM dutasteride (DU). Lane 12, Cell refers to HaCaTs. Lanes 12−16, testosterone (T) standards at different concentrations. The concentrations of compound **4** were shown at the bottom of each lane in μM. (**b**) The inhibition of SRD5A1 activity and HaCaTs viability in the treated cells. The half-maximal inhibitory concentration (IC_50_) of compound **4** against SRD5A1 activity was 1.44 ± 0.13 µM. The data of three independent experiments were represented as means ± SD. The viability and inhibitory of the compound-treated cells was compared to that of the untreated controls;^****^p < 0.0001.

Therefore, caffeic acid *N*-[3,5-bis(trifluoromethyl)phenyl] amide (compound **4**) was chosen for further study. Serial dilutions within the range of sub-cytotoxic concentrations were prepared for determining the compound’s effective inhibitory concentration. Its concentrations of 0.2, 0.5, 1, and 2.5 µM displayed no significant cytotoxicity between control and treatment groups. Compound **4** exhibited potent cell-based inhibition of DHT production with IC_50_ at 1.44 ± 0.13 µM (Fig. 5). Although the viability of the treated cells was lower than 80% at 5 µM, compound **4** demonstrated concentration-dependent inhibition of DHT production at 0.2–2.5 µM.

Notably, the observed DHT inhibition by compound **4** was 3 times more potent than that of avicequinone C, a potential steroid 5α-reductase inhibitor found to exhibit IC_50_ at 4.45 ± 0.42 µM using the same HaCaT cell-based assay^17^. Thus, caffeic acid *N*-[3,5-bis(trifluoromethyl)phenyl] amide (**4**) would be a promising non-steroidal modulator for SRD5A1, which led to the reduction of DHT production. Furthermore, compound **4** composes of three scaffolds, including a 3,4-dihydroxycinnamoly group, amide core, and *N*-[3,5-bis(trifluoromethyl)phenyl side chain, that could play a specific role in its mechanism of inhibition.

### Compound *4* modulates SRD5A1 protein expression

**Figure 6:**
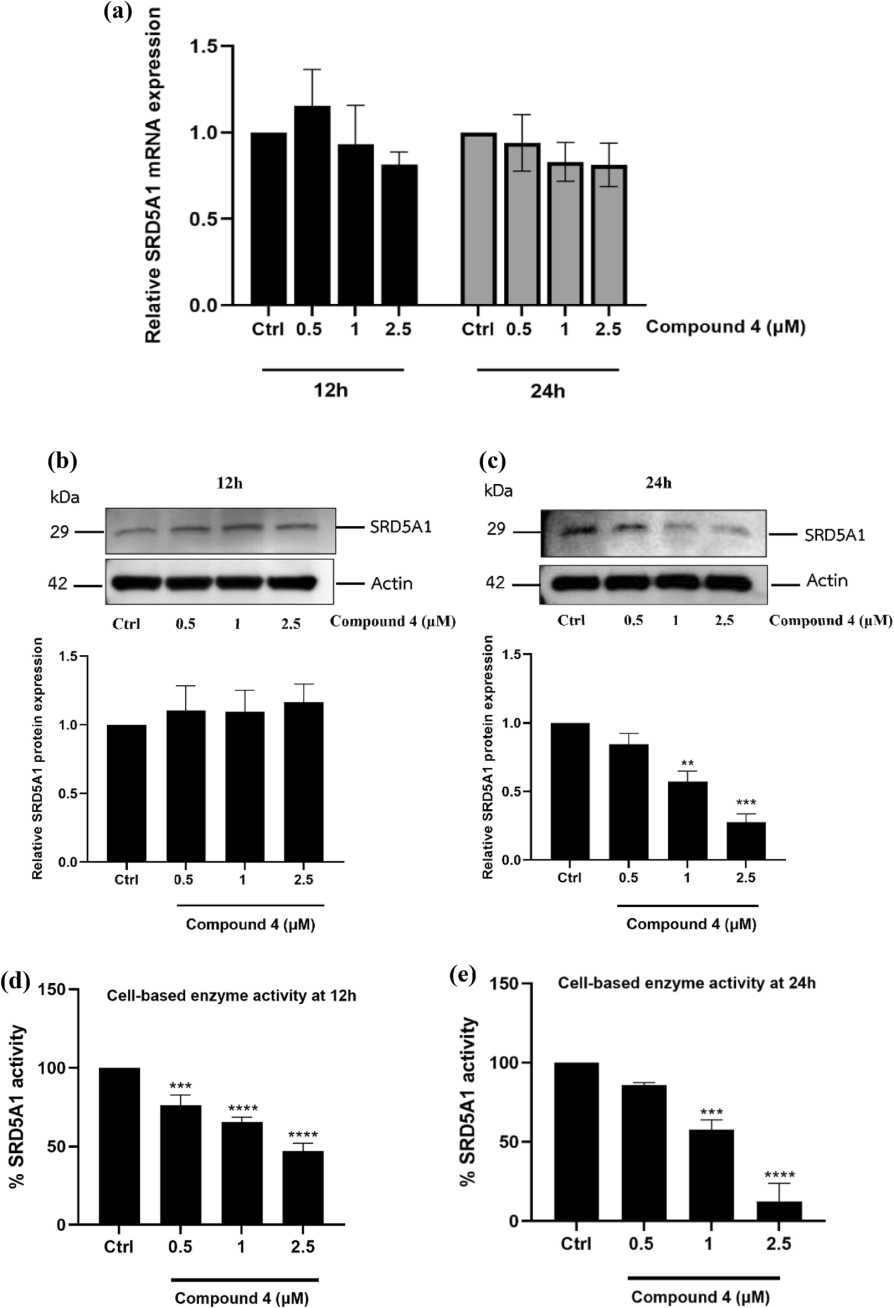
Expression levels of SRD5A1 mRNA and protein after treatment with various concentration of compound **4** (0 − 2.5 µM) as relative of the untreated control cells at 12 h and 24 h. (**a**) Expression level of SRD5A1 mRNA at 12 h and 24 h. The result mRNA expression was represented as Target gene/GAPDH and was analyzed via qRT-PCR. (**b**) Expression level of SRD5A1 protein at 12 h. (**c**) Expression level of SRD5A1 protein at 24 h. (**d**) SRD5A1 enzyme activity at 12 h (**e**) SRD5A1 enzyme activity at 24 h. All data were obtained in triplicate experiments and expressed as the mean ± SEM. **p < 0.001, ***p < 0.0005, ****p < 0.0001 versus untreated control cells (Ctrl).

We found that HaCaT cells clearly expressed the SRD5A1 gene (Fig. 3). The underlying mechanism of compound **4** was further examined by focusing on mRNA and SRD5A1 expression. HaCaT cells were treated with compound **4** at various concentrations of 0.5, 1, and 2.5 µM for 12 h and 24 h. The results of real-time RT-PCR showed that compound **4** did not affect the mRNA expression of SRD5A1 at both incubation times (Fig. 6a). This result suggested that the inhibition of compound **4** was independent of SRD5A1 gene expression. Next, the level of protein expression by western blot analysis was observed. Western blot results showed a significant decrease in SRD5A1 protein expression at 1 and 2.5 µM of compound **4**-treated HaCaT cells relative to the expression in the control at 24 h (Fig. 6c), whereas there were no significant changes in the level of SRD5A1 protein at 12 h (Fig. 6b). SRD5A1 enzyme activities at 12 h and 24 h were also compared. Treating HaCaT cells with compound **4** at various concentrations for 12 h showed approximately 50% inhibition at 2.5 µM of compound **4** (Fig. 6d), whereas the expression levels of both mRNA and SRD5A1 did not change. This finding suggested that compound **4** could modulate SRD5A1 functions according to the reduction of testosterone to DHT through a direct enzyme suppression mechanism that tended to occur rapidly. However, our results at 24 h clearly demonstrated that compound **4** at 2.5 µM suppressed the expression level of SRD5A1 protein > 50% relative to the expression in untreated control cells in a dose dependent manner. The protein suppression results corresponded with a decrease in the activity of SRD5A1 based on a HaCaT cell-based assay with direct DHT detection by HPTLC (Fig. 6e). Thus, the possible modulatory mechanism of compound **4** involves the suppression of both direct enzymatic function and expression of SRD5A1.

### Whole-cell-based kinetic study of HaCaT-produced SRD5A1 treated with compound *4*

As part of a kinetic study of SRD5A1 in human keratinocyte cells, we first performed a kinetic investigation to determine the inhibition rate of compound **4** against HaCaTs-produced SRD5A1. The enzyme source was prepared from HaCaT cells extracted in lysis buffer containing 1% protease and phosphatase inhibitors in Tris–HCl buffer pH 7.4. The lysate cells were homogenized and centrifuged. After the supernatant was collected, the enzyme reactions were examined using freshly prepared crude SRD5A1 (1000 μg of total protein), testosterone (10 µM), dithiothreitol (40 mM), and NADPH (100 µM)^36,37^. Then, the reactions were incubated and the enzyme activities at 30, 60, 90, 120 and 180 min were evaluated by direct DHT detection by HPTLC. Attempts to purify the crude extracted enzyme on a PD-10 desalting column (Cytiva 17-0851-01, Sigma-Aldrich, Singapore) and Amicon Ultra 3 k (C7115, Merck, Darmstadt, Germany) were performed. The obtained enzyme was then used in the enzyme kinetic reaction as previously mentioned. However, our kinetic studies using enzyme-based and direct DHT detection by HPTLC failed, probably because of the instability of SRD5A1 at the pHs used in the purified process^38,39^. (See Supporting Information for HPTLC chromatograms, Figs. S11−S12).

**Figure 7:**
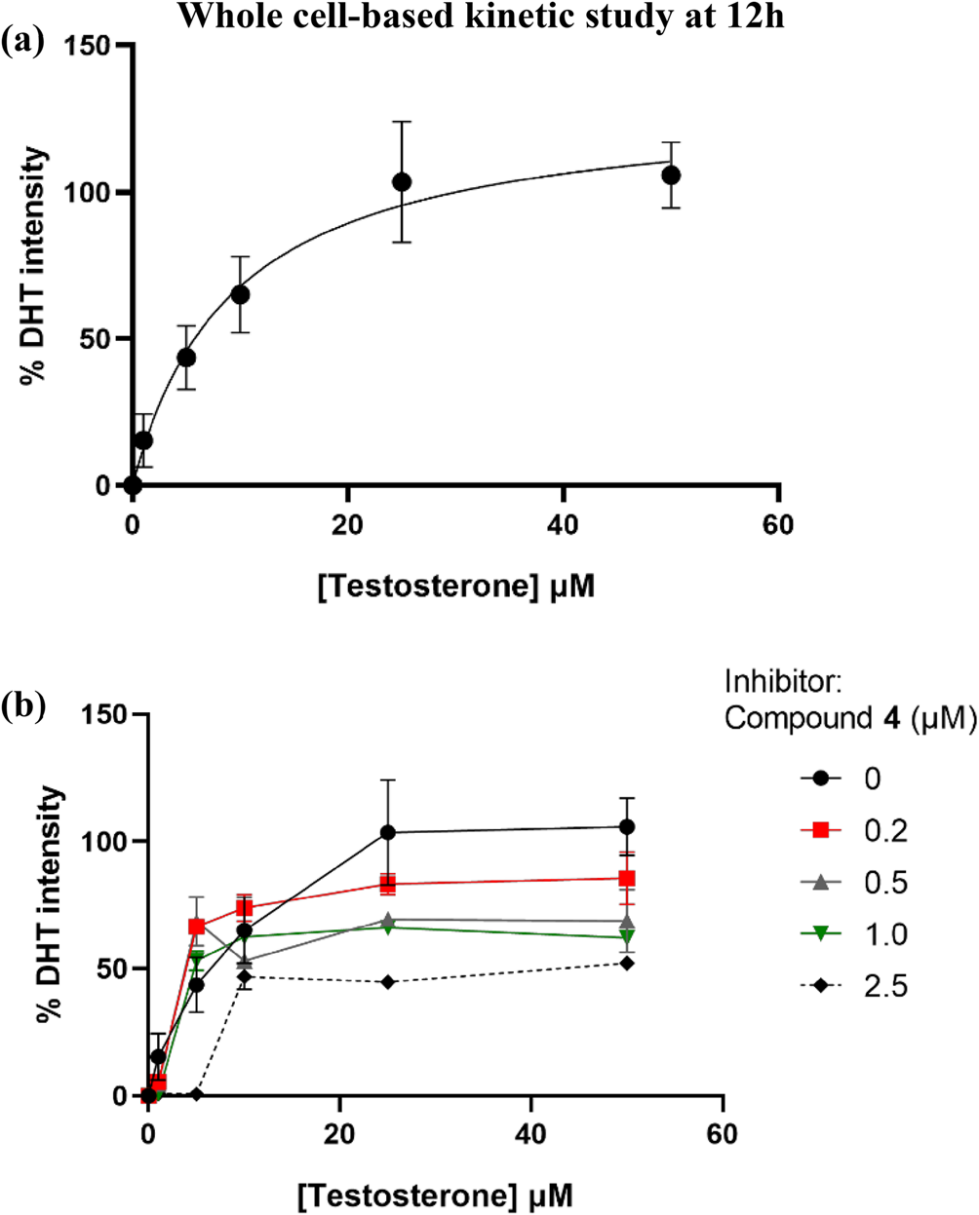
Whole-cell-based kinetic study of compound **4** on the inhibition of SRD5A1 in HaCaT cells at 12 h. (**a**) % dihydrotestosterone (DHT) intensity in variation of testosterone without compound **4** as an inhibitor. (**b**) Rate of DHT formation in variation of testosterone (0 − 50 µM) and compound **4** (0 − 2.5 µM) as an inhibitor. All data were obtained in triplicate experiments and expressed as the mean ± SEM.

**Figure 8:**
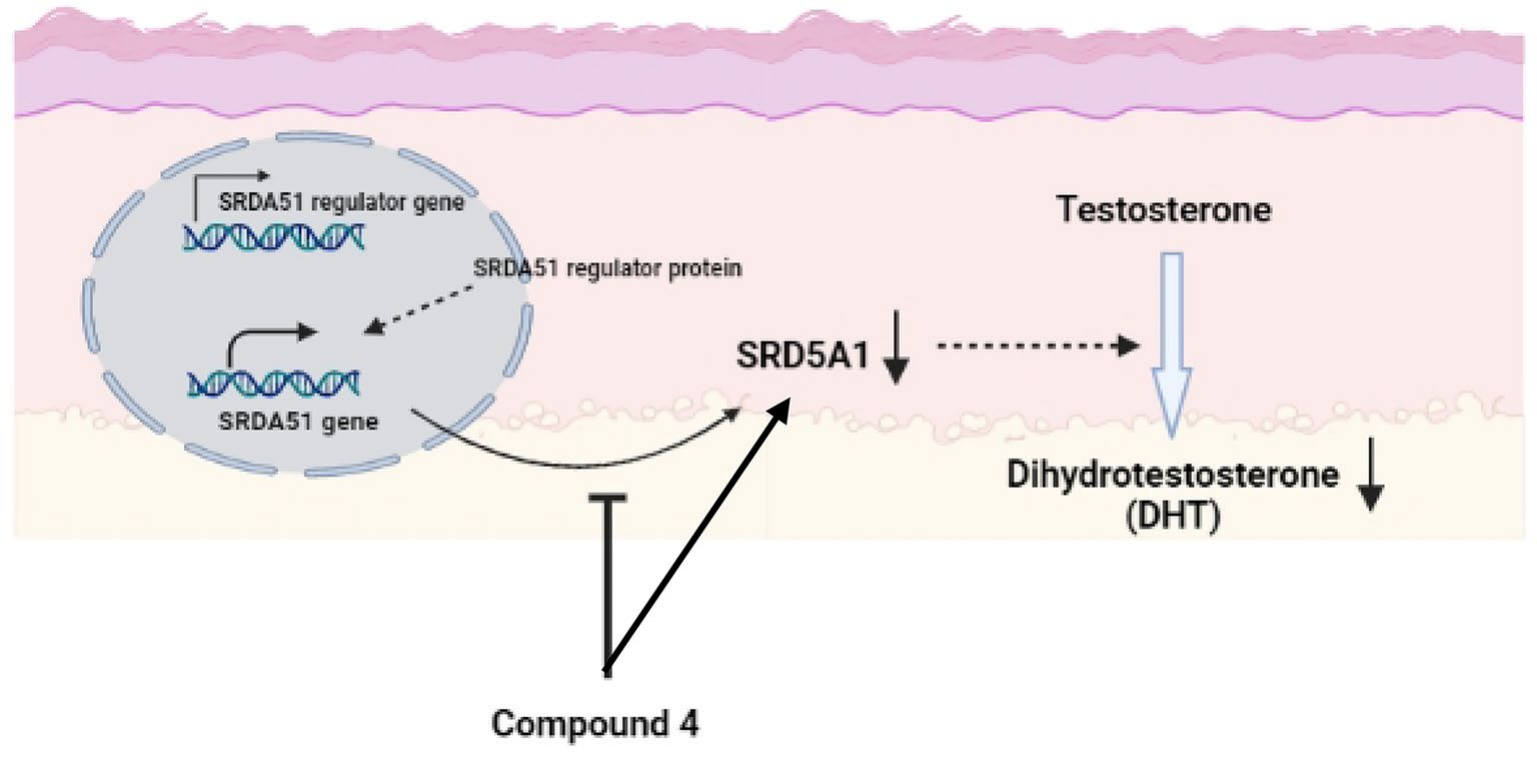
Schematic diagram demonstrating a possible mechanism of compound **4** on the modulation of SRD5A1 in human keratinocyte cells. Compound **4** mediates the lower level of DHT production together with down-regulating of SRD5A1 protein expression, which possibly involves the mixed-mode inhibition.

After the unsuccessful direct SRD5A1 enzyme-based kinetic study, a whole-cell-based kinetic study^40^ using an HaCaT cell-based assay was investigated with direct DHT detection by HPTLC for the first time. Under cell-based conditions, HaCaTs-produced SRD5A1 smoothly converted testosterone to DHT at the amount detected by the HPTLC technique. Initially, the reduction of testosterone to DHT by HaCaTs-produced SRD5A1 was evaluated. Whole cells were treated with various concentrations of substrate, testosterone (0 − 50 µM) for 12 h, and the cell viabilities were approximately 80%. The formation of the enzymatic product, DHT, was clearly shown by HPTLC (Fig. S13). The whole-cell inhibitory activities in the presence of substrate, testosterone (10 µM), and compound **4** (2.5 µM) as the inhibitor were investigated at 6 h, 12 h, and 24 h (Figs. S14−S16). Then, HaCaT cells were treated with various concentrations of both substrates, testosterone (0−50 µM) and compound **4** (0−2.5 µM) as the inhibitor (Figs. S17−S20 and Tables S1−S2). Low enzyme activities were observed for < 6 h. At 12 h; the enzyme activity appeared to reach a maximum of 80% cell viability, whereas at 24 h with a high concentration of testosterone, the cell viability was < 80%. Thus, the HaCaT cell-based kinetic study was performed with a 12-h of incubation time. The densitometric values were used to determine the kinetic rate and calculate the whole-cell K_m_ of testosterone. The results showed that the DHT intensity increased in a dose-dependent manner (Fig. 7a). The kinetic constant was based on the non-linear analysis and the Michaelis–Menten method gave a whole-cell K_m_ value of 9.287 µM. Previous studies have shown a testosterone affinity, with K_m_ value ranging from 1 to 5 µM in other cell lines, including dermal papilla, prostate cancer, and sebaceous glands^39,41,42^. Non-linear modeling of experimentally evaluated V_max_ in the absence of inhibitors gave a V_max_ value of 130.9. The whole-cell K_m_ of testosterone was almost equal to the concentration of testosterone used in our HaCaTs-produced SRD5A1 inhibitory assay wherein 10-µM testosterone was used. The inhibition of compound **4** on SRD5A1 based on direct DHT detection by HPTLC analysis enabled calculation of the inhibitory rate constant (K_i_) by the non-linear analysis after treatment with various concentrations of both compound **4** and testosterone. The estimated whole-cell K_i_ value for compound **4** was 2.382 µM determined by mixed-model inhibition analysis. The inhibition profile is shown in Fig. 7b. As the results in Table 2 show, adding compound **4** as an inhibitor at concentrations of 0.2 − 1.0 µM caused a decline in both V_max_ and K_m_. At this concentration range, compound **4** could become an enzyme activator^43^ or uncompetitive inhibitor, with compound **4** binding to the enzyme-NADP + complex after DHT leaves^1^. The alteration of V_max_ and K_m_ revealed a possible mixed inhibition mechanism of compound **4**. Interestingly, compound **4** exhibited potent inhibition at 2.5 µM, a concentration that is close to the whole-cell K_i_ value of 2.382 µM. In addition, adding compound **4** at a concentration of 2.5 µM decreased the V_max_ and increased K_m_ causing enzyme inhibition^43^ because of the competitive mechanism wherein compound **4** binds the enzyme-NADPH complex. Therefore, the results of the expression levels of SRD5A1 mRNA and protein along with the kinetic studies obtained from the whole HaCaT cell-based assay with non-radioactive and direct DHT detection by HPTLC detection suggested that compound **4** modulates SRD5A1 via the dual actions that affect the level of SRD5A1 protein expression and the activity of the SRD5A1 enzyme (Fig. 8).

**Table 2:** Comparison of apparent V_max_ and K_m_ in the variation of compound **4**.

Entry	Compound **4** (μM)	V_max_	K_m_ (μM)
1	0	130.9	9.287
2	0.2	96.08	3.534
3	0.5	76.27	2.735
4	1.0	74.06	3.305
5	2.5	69.85	13.81

V_max_ and K_m_ were determined by using Michaelis–Menten equation.

### Binding of compound *4* to SRD5A1 in molecular docking analysis

Compound **4** at 2.5 µM showed significant suppression of SRD5A1 function and expression, whereas the expression level of SRD5A1 protein remained the same at 12 h. This finding together with the whole-cell-based kinetic study against SRD5A1 at 12 h suggests that compound **4** at a concentration of 2.5 µM could inhibit SRD5A1 directly by competitive inhibition. Therefore, molecular docking and molecular dynamics were investigated to obtain insightful information regarding the binding interactions and stability between compound **4** and the SRD5A1 active site. Because of the lack of a crystal structure for human SRD5A1, the AlphaFold-generated SRD5A1 structure was used as the surrogate protein for our experiment^44^. Comparison of the structural similarities between the AlphaFold-generated SRD5A1 and the recently reported human SRD5A2^35^ showed that both enzyme structures have whole structure and binding-site similarities of 66% and 78%, respectively (Fig. S21). Regarding the conserved steroid 5α-reductase catalytic sites of both SRD5A1 and SRD5A2, the key binding site of SRD5A1 involves E60, Y95, and M119. These amino acid residues are located at the corresponding sequence of SRD5A2 as E57, Y91, and R114, respectively^34^. Noted that, a successful prediction is considered the RMSD value from the comparison of the docked binding and the model is < 2.0 Å^45^. In the system validation of the GOLD function by redocking studied, we reliably reproduced the binding mode, as the RMSD values were 1.65 and 1.28 Å for SRD5A1 and SRD5A2, respectively (Fig. S22). Therefore, our model could be acceptable for study.

**Figure 9:**
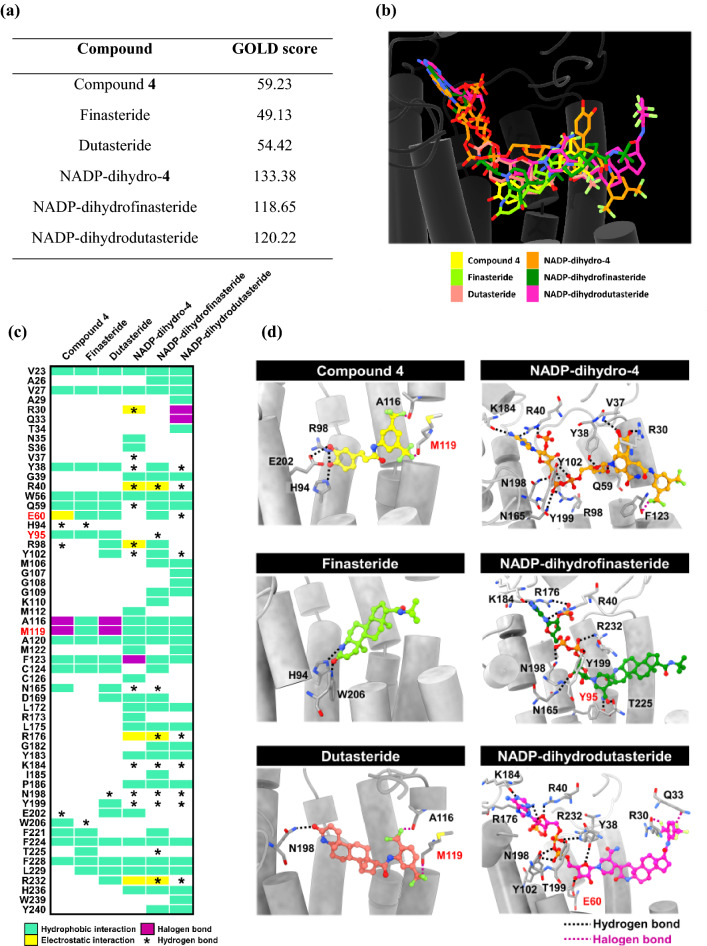
Binding interactions of the caffeic acid *N*-[3,5-bis(trifluoromethyl)phenyl] amide derivative (compound **4**) and known inhibitors, finasteride and dutasteride with human SRD5A1 compared with their NADP adducts. (**a**) GOLD docking scores. (**b**) Superimposition of all the docked compounds. (c) The heat map of inhibitor-protein interaction is based on the default of the Discovery Studio Visualizer 2.5, which is determined according to the distance criteria of nonbonded interactions as follows: (1) hydrophobic interactions are derived from van der Waals fraction (≤ 0.70 Å), amide-π stacked (≤ 4.50 Å), alkyl (≤ 5.50 Å), π-alkyl (≤ 4.0 Å), π-sigma (≤ 4.0 Å) and π-π T-shaped (π-π centroid ≤ 6.0 Å); and (2) electrostatic interactions include π-anion (≤ 5.0 Å), π-cation (≤ 5.0 Å), salt bridge and attractive charge (≤ 5.6 Å). Additionally, the distances related to hydrogen bond (H-bond donor–-acceptor) and halogen bond (fluorine) are ≤ 3.40 Å and ≤ 3.70 Å, respectively. The red color texts represent the key catalytic residues. (d) The 3D geometry of all the docked compounds in the binding site with indicated hydrogen bond (black) and halogen bond (magenta) interactions.

Next, we investigated the binding interaction, inhibitory mechanism, and efficacy of compound **4** toward SRD5A1 using molecular docking with the GOLD program (Fig. 9). In addition, compound **4** with a covalently bound NADP adduct (NADP-dihydro-**4**; Fig. S23) was investigated in comparison with known steroid 5α-reductase inhibitors, finasteride and dutasteride, in the same form.

The resultant GOLD scores (Fig. 9a) showed that the binding efficiency with steroid 5α-reductase of the free-form compound **4** (59.23) was higher than finasteride (49.13) and dutasteride (54.42). A covalent formation with an NADP adduct could significantly strengthen compound **4**’s binding (133.38) relative to that of dutasteride (120.22), whereas a redocked NADP-dihydrofinasteride obtained a slightly lower binding affinity (118.65). In this study, 5α-reductase inhibition was relatively higher for dutasteride (inhibitor of both SRD5A1 and SRD5A2) than finasteride ***(***a specific inhibitor of SRD5A2***)***, consistent with previous reports^34^. Notably, compound **4** in both free form and covalently bound form occupied the binding pocket of SRD5A1 in an orientation similar to the NADP-dihydrofinasteride adduct (Fig. 9b). The binding of compound **4** and NADP-dihydro-**4**, to steroid 5α-reductase involved 20 and 37 residues, respectively (Fig. 9c). Free-form compound **4** hydrophobically interacted with steroid 5α-reductase similar to those with finasteride and dutasteride, including V23, V27, Y38, W56, Q59, Y95, A120, F123, C124, F224, and F228. Interestingly, the trifluoromethyl phenyl group of the amide side chain of compound **4** could interact with A116 and the catalytic residue M119 via halogen bonds similar to dutasteride (Fig. 9c and d). In addition, three more hydrogen bonds with H94, R98, and E202 of SRD5A1 were formed with the hydroxyl group on the 3,4-dihydroxycinnamoyl moiety. The catalytic residue E60 electrostatically stabilized the binding of compound **4** instead of forming hydrogen bonding in most of the other compounds. Notably, halogen bonding can be found in the complex of the NADP-dihydrodutasteride adduct and the enzyme as well as NADP-dihydro-**4**. As expected, more ligand–protein interactions were observed to stabilize NADP-dihydro-**4**. Additional hydrophobic interactions with residues N35, S36, G39, M112, M122, C126, D169, L172, R173, L175, Y183, P186, L229, and H236 and electrostatic attractions with the five charged residues, R30, R40, R98, R176, and R232, were observed. Furthermore, hydrogen bond formation between NADP-dihydro-**4** and steroid 5α-reductase was also increased in the (1) adenine ring with R40 and K184; (2) 3,4-dihydroxycinnamoyl scaffold with R30 and V37; and (3) phosphate groups with R98, Y102, N165, N198, and Y199; and (4) ribose sugar with Y38 and Q59.

Many residues in SRD5A1, such as V23, V27, G39, W56, A116, M119, A120, L172, L175, Y183, P186, F224, F228, L229, and H236, could hydrophobically interact with NADP-dihydrofinasteride and NADP-dihydrodutasteride, so NADP-dihydro-**4** could interact with these amino acids as well. NADP-dihydro-**4** could form 11 hydrogen bonds with SRD5A1, similar to the number of bonds formed between the enzyme and both inhibitors, finasteride and dutasteride (nine bonds). In addition, the essential amino acids in the catalytic pocket, E60, Y95, and M119, have been detected. Both E57 and Y91 were reported as the crucial hydrogen bond sites for the enzymatic reduction of testosterone to DHT using NADPH as a cofactor in steroid 5α-reductase^34,35^. Therefore, E60 and Y95 were also important residues in SRD5A1. Although, the NADP-dihydro-4 adduct demonstrated no hydrogen bonding with these essential amino acids in the catalytic pocket. However, hydrogen bonds with any amino acids could be formed at the surrounding residues as mentioned above, thereby preventing the substrate from reacting in this position. In contrast, the NADP-dihydrofinasteride and NADP-dihydrodutasteride adducts showed only hydrogen bond interactions with the key catalytic residues Y95 and E60, respectively. Notably, these findings on the hydrogen bond interactions of the NADP-dihydro-**4** adduct with R40, K184, N198, and Y199 are correlated with the results of both NADP-dihydrofinasteride and NADP-dihydrodutasteride adducts. Furthermore, compound **4** contains a trifluoromethylbenzene moiety similar to the reported 5α-reductase inhibitors, including CHEMBL2115222 for SRD5A1^46^ and CHEMBL35176, CHEMBL36022 and CHEMBL36688 for SRD5A2^47^ and dutasteride. Compound **4** exhibited good drug-like physicochemical properties (Table S3). Therefore, compound **4** is predicted to be an inhibitor of SRD5A1 and could potentially be further developed into a novel antiandrogen agent.

### Molecular dynamics study of compound *4* against SRD5A1

**Figure 10:**
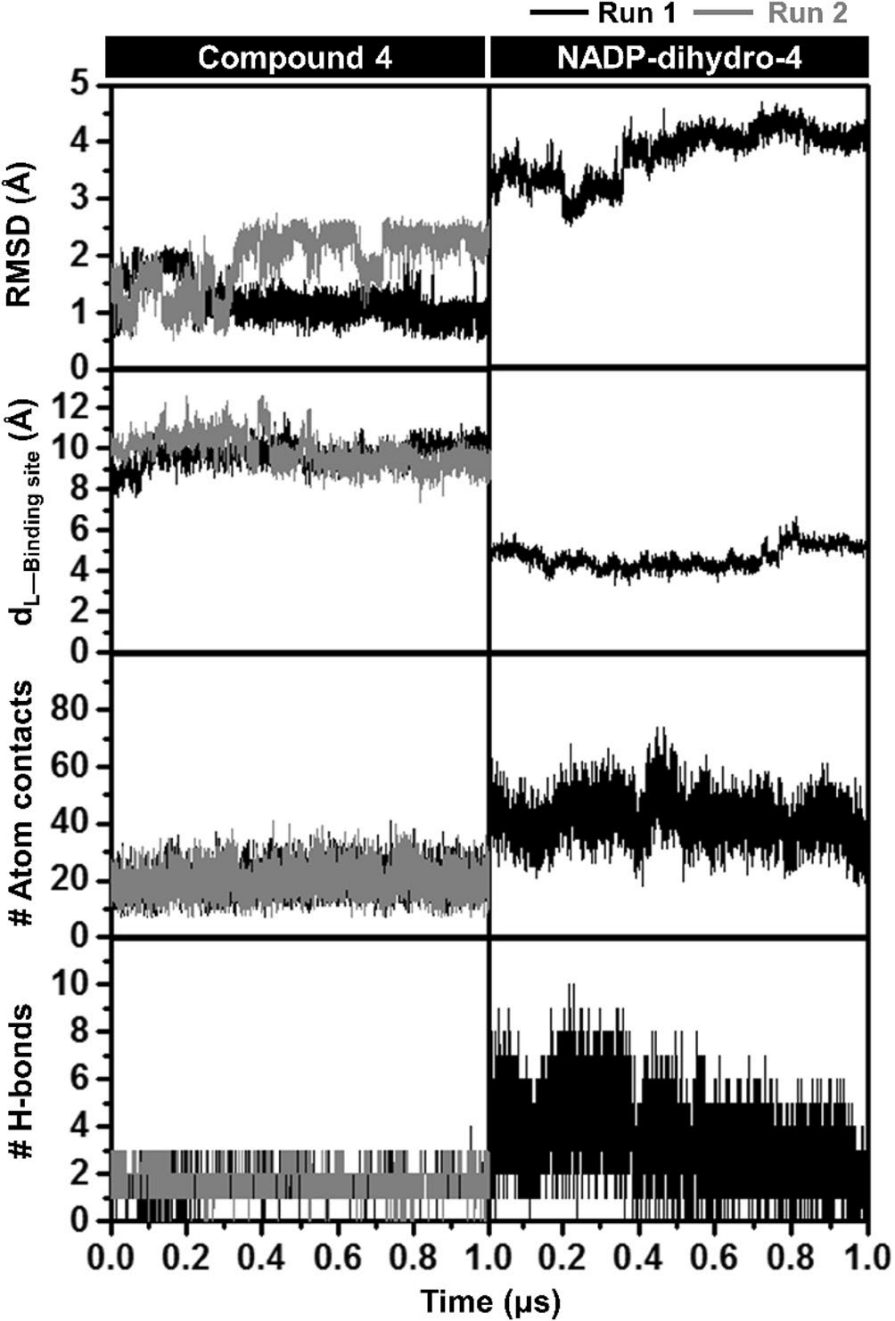
The plots of the RMSD of compound **4** and NADP-dihydro-**4**, the distance measured between the centers of mass of caffeic acid *N*-[3,5-bis(trifluoromethyl)phenyl] amide derivative (compound **4**)/NADP-dihydro-**4** and binding residues (d_L-Binding site_), # Atom contacts and #H-bonds of compound **4** (Runs 1 and 2) and NADP-dihydro-**4** (Run 1) in complex with SRD5A1 along with the 1-μs MD simulations. The angle between the hydrogen bond donor (HD) and hydrogen acceptor (HA) were employed as a criterion for strong hydrogen bond calculations of NADP-dihydro-**4** system, with the distance and angle of ≤ 2.8 Å and ≥ 150°, respectively.

SRD5A1 bound with the potential compound **4** (Runs 1 and 2), and NADP-dihydro-**4** (Run 1) in the phospholipid bilayer was investigated by all-atom MD simulations for 1 μs. The three following interpretations along with the simulation time in Fig. 10 were used to consider the system stability: (1) the root-mean-square deviation (RMSD) of atomic positions of compound **4** and NADP-dihydro-**4** adduct; (2) the distance between the centers of mass of the caffeic acid *N*-[3,5-bis(trifluoromethyl)phenyl] amide derivative and the binding residues including the catalytic triad (E57, Y91, and R114) and the surrounding residues within 3.5 Å (V23, A26, R40, W56, Q59, A116, A120, M122, F123, L172, L175, R176, T181, G182, Y183, K184, P186, N198, Y199, F224, F228, R232, H236, W239, and Y240) (d_L-binding site_); (3) # atom contacts within 3.5 Å sphere of ligand; and (4) # H-bonds formed between the compound and target protein. The last one was calculated using two different conditions. The distance and angle between the hydrogen bond donor (HD) and hydrogen acceptor (HA) of ≤ 2.8 Å and ≥ 150° were used to determine strong H-bonds, while those of ≤ 3.5 Å and ≥ 120° were applied for counting all possible H-bonds. The obtained results from the last 100 ns were selected for discussion.

The distance plot in Fig. 10 showed that both compound **4** and its adduct were stably within the active site (~ 9.7 and ~ 5.4 Å, respectively) as seen by the snapshots per time in Fig. S24 and the movies generated from Run 1 (Videos S1–S2). The trajectory files of each complex from the Run 1 system are given in Files S1–S4. Consistent with the docking study, the atom contacts and H-bonds of the adduct interacted with the SRD5A1 significantly better than compound **4 **(Figs. 10 and S25). Noted that, the other independent MD simulations systems (0.7 μs for Run 3 of compound **4**, and 0.5 μs for Runs 2 and 3 of NADP-dihydro-**4**) showed a similar pattern (Figs. S26–27). This finding was supported by the binding affinity of compound **4** and NADP-dihydro-**4** predicted using the end-point binding free-energy calculations with the MM/(PB)GBSA methods in Table 3. The NADP-dihydro-**4** complex provided the binding free energy of − 36.58 ± 0.14 kcal/mol for MM/PBSA and − 15.02 ± 0.14 for MM/GBSA, which was relatively lower than that of the complex of free compound **4** (− 9.00 ± 0.72 and − 3.95 ± 0.72 kcal/mol for MM/PBSA and MM/GBSA, respectively). Although the electrostatic interaction was found to be the main force for complexation, particularly the adduct (${\Delta E}_{\mathrm{ele}}$ of − 1213.59 ± 5.53 kcal/mol), it was compensated by a large contribution of the polar solvation term (${\Delta G}_{\mathrm{sol}}^{\mathrm{ele}}$ of 615.98 ± 2.16 kcal/mol and 1243.13 ± 4.88 for PB and GB).

**Table 3:** Binding free energy and energy components (kcal/mol) of compound **4** (Runs 1 and 2) and NADP-dihydro-**4** (Run 1) in complex with SRD5A1 calculated with MM/(PB)GBSA method from the last 100-ns MD simulations.

Energy component	Compound 4	NADP-dihydro-4
**Gas term**		
${\Delta E}_{\mathrm{vdW}}$	− 27.40 ± 0.47	− 74.91 ± 0.52
${\Delta E}_{\mathrm{ele}}$	− 55.62 ± 0.76	− 1213.59 ± 5.53
${\Delta E}_{\mathrm{MM}}$	− 83.02 ± 0.64	− 1288.50 ± 5.55
-*T*Δ*S*	21.96 ± 1.39	41.45 ± 2.20
**Solvation term**		
${\Delta G}_{\mathrm{sol}(\mathrm{MM}/\mathrm{PBSA})}^{\mathrm{ele}}$	30.17 ± 0.31	615.98 ± 2.16
${\Delta G}_{\mathrm{sol}(\mathrm{MM}/\mathrm{GBSA})}^{\mathrm{ele}}$	62.79 ± 0.59	1243.13 ± 4.88
${\Delta G}_{\mathrm{sol}(\mathrm{MM}/\mathrm{PBSA})}^{\mathrm{nonpolar}}$	− 5.89 ± 0.02	− 12.31 ± 0.03
${\Delta G}_{\mathrm{sol}(\mathrm{MM}/\mathrm{GBSA})}^{\mathrm{nonpolar}}$	− 5.66 ± 0.03	− 11.10 ± 0.04
${\Delta G}_{\mathrm{sol}(\mathrm{MM}/\mathrm{PBSA})}$	24.28 ± 0.31	603.67 ± 2.15
${\Delta G}_{\mathrm{sol}(\mathrm{MM}/\mathrm{GBSA})}$	57.13 ± 0.57	1232.03 ± 4.86
**Binding free energy**		
${\Delta G}_{\mathrm{bind}(\mathrm{MM}/\mathrm{PBSA})}$	− 9.00 ± 0.72	− 36.58 ± 0.14
${\Delta G}_{\mathrm{bind}(\mathrm{MM}/\mathrm{GBSA})}$	− 3.95 ± 0.72	− 15.02 ± 0.14

Data are shown as means ± the standard error of the mean (SEM).

**Figure 11:**
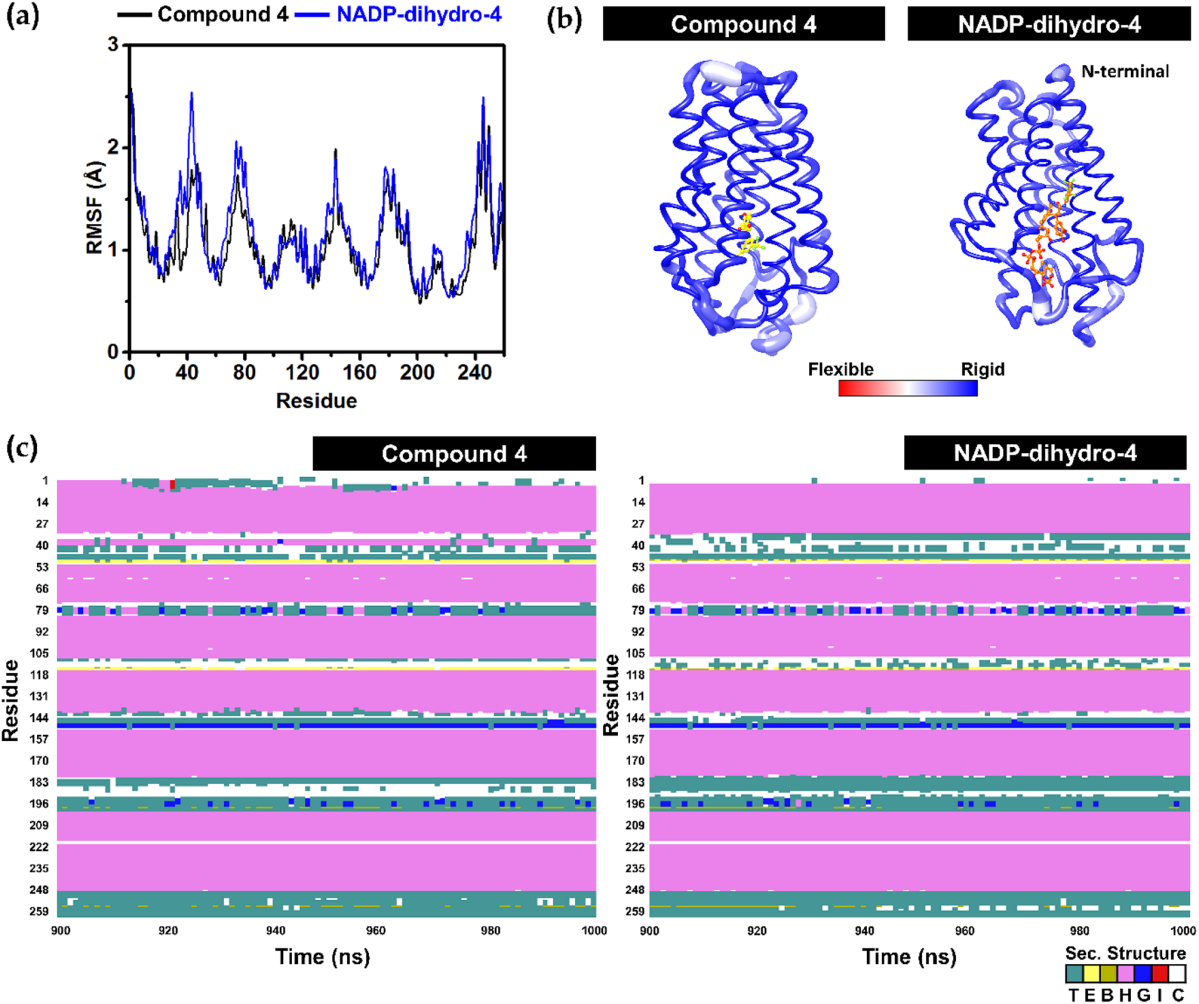
Structural investigations of the SRD5A1 in complex with compound **4** and NADP-dihydro-**4** along with the MD simulations (Run 1). (**a**) RMSF and (**b**) B-factor of SRD5A1 protein. (**c**) Secondary structure timeline analysis as computed by VMD, where the turn (T) and extended conformation (E) are represented in teal and yellow, respectively; isolated bridges are in dark yellow; α-helix is in pink (H), blue (3–10 helix) and red (π-helix); and random coils are in white.

**Figure 12:**
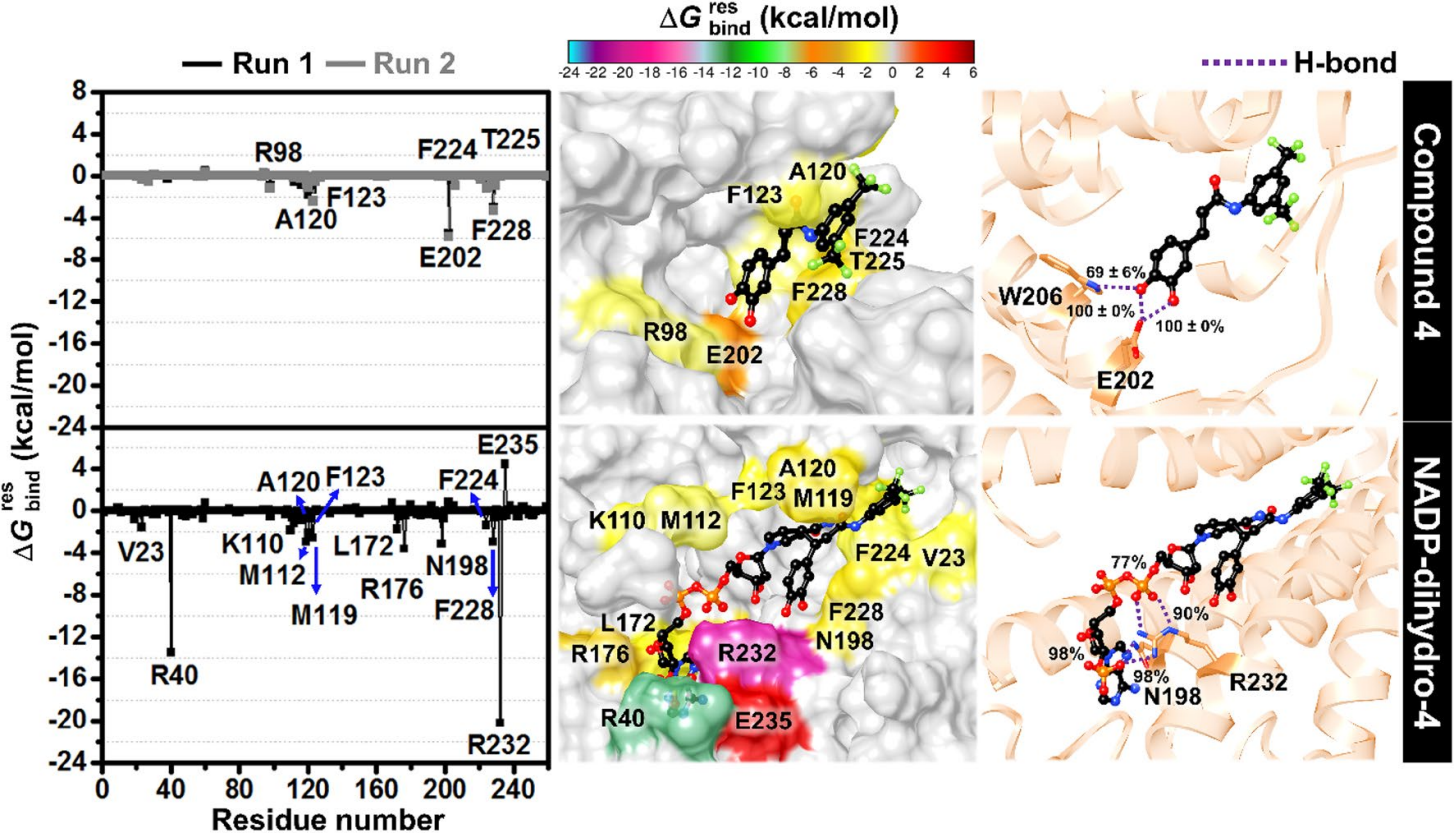
Per-residue decomposition free energy of the SRD5A1 in complex with compound **4** (Average data from Runs 1 and 2) and NADP-dihydro-**4** (Run 1) and hydrogen bond occupation (cut off > 65%) from the last 100 ns MD simulation. The lowest and highest energies range from cyan to dark red, respectively. The representative frame of the most frequent binding of each complex resulted from RMSD clustering using Chimera 1.15^48^ is given.

Furthermore, the protein structural investigations of SRD5A1 in complex with compound **4** and NADP-dihydro-**4** in terms of (1) the root-mean-square fluctuation (RMSF), (2) B-factor and (3) secondary structure analysis were investigated and presented in Fig. 11. We found that in both complexes the N-terminal and turn conformations of 5α-reductase were quite flexible, whereas the α-helix structures were relatively stable (Fig. 11a and b). Therefore, the two compounds could interact well and stabilize within the SRD5A1 embedded in the lipid bilayer. These findings were supported by the secondary structure per time analysis in which the protein retained its conformation throughout the MD simulations (Fig. 11c). Additionally, the key binding residues of inhibitors were evaluated based on MM/GBSA method (Fig. 12). The energy contributions of SRD5A1 residues <  − 1.0 kcal/mol are considered for discussion. We found that compound **4** could be stabilized within the pocket by interacting with R98, A120, F123, E202, F224, T225 and F228 residues. While NADP-dihydro-**4** bind better to SRD5A1 with additional interactions to the 9 residues including V23, R40, K110, M112, L172, R176, N198, F224 and R232. Interestingly, the trifluoromethyl phenyl group of the amide side chain of NADP-dihydro-**4** hydrophobically interact with the catalytic residue M119. The binding of the NADP-dihydro-**4** to the V23, R40, A120, A120, F123, R176, N198, F224, F228 and R232 showed a similar result as the docking study. Besides, the hydroxyl group on the 3,4-dihydroxycinnamoyl moiety of compound **4** forms hydrogen bonds with E202 (100 ± 0% and 100 ± 0%) and W206 (69 ± 6%). Whereas hydrogen bond formation between NADP-dihydro-**4** and SRD5A1 was increased in the (1) adenine ring with N198 (98%) and (2) phosphate group with R232 (98, 90 and 77%).

## Materials and methods

### Chemicals and general procedures

Commercial caffeic acid (Sigma Life Science) was stored at 0 °C until use. All other commercial reagents were from TCI chemicals. Anhydrous solvents were dried over 4 Å molecular sieves. All reactions were performed in oven-dried glassware and magnetically stirred under an inert atmosphere at room temperature unless otherwise described. In addition, all reactions were monitored by thin-layered chromatography (TLC) using an aluminum silica gel 60 F254 (Merck). Flash column chromatography was carried out using silica gel as the stationary phase. Additionally, all solvents, such as methanol, ethyl acetate, dichloromethane, and hexane, were purified by distillation before use. Moreover, infrared (IR) spectra were measured on a Perkin Frontier Fourier Transform Infrared Spectrometer. Furthermore,^1^H and^13^C nuclear magnetic resonance (NMR) spectra were obtained on a Bruker ADVANCE NEO 400 MHz NMR spectrometer, and deuterated methanol (CD_3_OD) served as the internal standard for both^1^H (3.31 and 4.78 ppm) and^13^C (49.15 ppm) spectra. Accurate mass spectra were obtained using an Agilent 6540 UHD Q-TOF LC/MS spectrometer. HPTLC was performed using a Merk silica gel 60 F254 glass HPTLC plate. All HPTLC mobile phases including cyclohexane (Sigma-Aldrich, Saint Louis, MO, USA), ethyl acetate (Merck, Darmstadt, Germany) and triethylamine (Merck, Darmstadt, Germany) were purchased. HPTLC instruments include a semi-automatic sampler Linomat 5, TLC Plate Heater, TLC Scanner 3, Visualizer 2 (CAMAG, Muttenz, Switzerland). Each chromatogram was captured and processed by HPTLC Software visionCATS 3.0 (CAMAG, Muttenz, Switzerland).

Human keratinocyte (HaCaT) (ATCC, Manassas, VA, USA) and DP cells (Applied Biological Materials Inc., Richmond, Canada) were obtained. In addition, Dulbecco’s Modified Eagle’s Medium (DMEM), fetal bovine serum (FBS), antibiotic–antimycotic, l-glutamine (GlutaMAX), sodium pyruvate, trypsin-ethylene diamine tetra-acetic acid, phosphate-buffered saline, Dulbecco’s phosphate-buffered saline, and 3-(4,5-dimethylthiazol-2-yl)-2,5-diphenyl tetrazolium bromide (MTT) (Thermo Fisher Scientific, Waltham, MA, USA) were purchased. Dimethyl sulfoxide (DMSO) was procured from Merck Millipore (Billerica, MA, USA).

### Synthesis of phenyl amide of caffeic acid (*2*)

Caffeic acid (**1**) (50 mg, 0.2775 mmol), 1-ethyl-3-(3-dimethyl aminopropyl) carbodiimide (EDCI^.^HCl; 53.2 mg, 0.2775 mmol), and hydroxybenzotriazole (HOBt; 42.5 mg, 0.2775 mmol) were weighed into an oven-dried round-bottomed flask and dissolved in dry tetrahydrofuran (5 mL). Then, an N_2_ gas balloon was used to create the inert atmosphere. The reaction mixture was mixed using a magnetic stir bar for 30 min at room temperature. Afterward, aniline (38 μL, 0.4163 mmol) was added to the mixture, and the reaction was stirred at room temperature for 24 h and monitored by TLC using an ethyl acetate:hexane solution (7:3 v/v) mixture as the mobile phase. Then, the volatile solvent was removed under reduced pressure, and the residue was redissolved in ethyl acetate (20 mL). The resulting solution was transferred to a separatory funnel and washed with water to remove the water-soluble by-products. Next, the organic layer was dried over anhydrous sodium sulfate, filtered, and evaporated under reduced pressure to produce a crude product. The crude product was purified using flash column chromatography with silica gel as the stationary phase and the ethyl acetate:hexane mixture as the eluent. Caffeic acid phenyl amide, or 3-(3,4-dihydroxyphenyl)-*N*-phenyl-2-propenamide (**2**), was obtained with an isolation yield of 71% (50.04 mg) as a yellow solid. IR (ATR, ν_max_ /cm) 3368.9 (br), 1735.2, 1655.2, 1597.0, 1525.9, 1498.9, 1441.4, 1352.5, 1281.5, 1183.2, 1112.1, 973.3, 848.2, 810.7, 754.4, 691.2, 562.0, and 456.9;^1^H − NMR (CD_3_OD, 400 MHz, δ in ppm) 7.64 (2H, dd, *J* = 8.0, 2.0 Hz, CH), 7.52 (1H, d, *J* = 15.6 Hz, = CH), 7.31 (2H, dd, *J* = 8.0, 7.2 Hz, CH), 7.10 (1H, d, *J* = 7.2 Hz, CH), 7.06 (1H, d, *J* = 2.0 Hz, CH), 6.95 (1H, dd, *J* = 8.0, 2.0 Hz, CH), 6.79 (1H, d, *J* = 8.0 Hz, CH), 6.56 (1H, d, *J* = 15.6 Hz, = CH);^13^C − NMR (CD_3_OD, 100 MHz, δ ppm) 167.52, 149.15, 146.90, 143.55, 140.24, 129.96 (3C), 128.39, 125.27, 122.52, 121.35, 118.84, 116.63, 115.31; HRMS m/z of caffeic acid phenyl amide was observed at 256.0969 ([M + H]^+^, calculated for C_15_H_13_NO_3_H, 256.0968).

### Synthesis of tert-butyl amide of caffeic acid (*3*)

Caffeic acid (**1**) (50 mg, 0.2775 mmol), 1-ethyl-3-(3-dimethylaminopropyl) carbodiimide (EDCI^.^HCl; 53.2 mg, 0.2775 mmol), and hydroxybenzotriazole (HOBt; 42.5 mg, 0.2775 mmol) were weighed into an oven-dried round-bottomed flask respectively and dissolved in dry tetrahydrofuran (5 mL). Then, an N_2_ gas balloon was used to create the inert atmosphere. The reaction mixture was mixed using a magnetic stir bar for 30 min at room temperature. Next, *tert*-butyl amine (44 μL, 0.4163 mmol) was added, and the reaction was stirred at room temperature for 24 h and monitored by TLC using an ethyl acetate:hexane solution (7:3 v/v) as the mobile phase. Then, the volatile solvent was removed under reduced pressure, and the residue was redissolved in ethyl acetate (20 mL). Next, the resulting solution was transferred to a separatory funnel and washed with water to remove the water-soluble by-products. Afterward, the organic layer was dried over anhydrous sodium sulfate, filtered, and evaporated under reduced pressure to produce a crude product. The crude product was purified using flash column chromatography with silica gel as the stationary phase and the ethyl acetate:hexane solution as the eluent. The *tert*-butyl amide of caffeic acid (**3**), or 3-(3,4-dihydroxyphenyl)-*N*-(1,1-dimethyl ethyl)-2-propenamide, was obtained with an isolation yield of 75% (48.67 mg) as a yellow solid. IR (ATR, ν_max_ /cm) 3228.7 (br), 2923.3, 1753.9, 1648.6, 1596.2, 1514.0, 1443.2, 1375.5, 1357.7, 1275.7, 1221.7, 1179.5, 1163.1, 1111.1, 1034.5, 974.5, 909.8, 856.7, 795.1, 779.1, 738.2, 567.7;^1^H − NMR (CD_3_OD, 400 MHz, δ in ppm) 7.54 (1H, d, *J* = 16.0 Hz, = CH), 7.03 (1H, d, *J* = 2.0 Hz, CH), 6.94 (1H, dd, *J* = 8.4, 2.0 Hz, CH), 6.77 (1H, d, *J* = 8.0 Hz CH), 6.24 (1H, d, *J* = 16.0 CH), 1.29 (9H, s, *tert*-butyl);^13^C − NMR (CD_3_OD, 100 MHz, δ in ppm) 169.87, 149.73, 147.08, 146.96, 127.81, 123.06, 116.62, 115.24, 114.96, 52.13, 30.59 (3C); HRMS m/z of *tert*-butyl amide of caffeic acid was observed at 236.1281 ([M + H]^+^, calculated for C_13_H_17_NO_3_H, 236.1281).

### Synthesis of N-[3,5-bis(trifluoromethyl)phenyl] amide of caffeic acid (*4*)

Caffeic acid (**1**) (50 mg, 0.2775 mmol), *N*,*N*′-dicyclohexylcarbodiimide (DCC, 46 µL, 0.2775 mmol), and 3,5-bis(trifluoromethyl)aniline (43 µL, 0.2775 mmol) were dissolved in dry tetrahydrofuran (5 mL) in an oven-dried round-bottomed flask. Then, the reaction mixture was refluxed for 3 h and monitored by TLC using an ethyl acetate: hexane solution (1:1 v/v) as the mobile phase. After TLC monitoring indicated completion, the reaction mixture was filtered to remove the solid by-products, concentrated, and purified by flash column chromatography using silica gel as the stationary phase and the ethyl acetate:hexane solution as the mobile phase. Caffeic acid *N*-[3,5-bis(trifluoromethyl)phenyl] amide (**4**), or 3-(3,4-dihydroxy phenyl)-*N*-[3,5-bis (trifluoromethyl) phenyl]-2-propenamide, was produced with the isolation yield of 77% (83.1 mg) as a pale-yellow powder. IR (ATR, ν_max_ /cm) 3399.9 (br), 2929.5, 2856.8, 1667.6, 1600.7, 1565.1, 1472.8, 1440.2, 1382.4, 1278.2, 1174.5, 1135.4, 975.6, 937.0, 886.6, 850.9, 811.0, 701.1, 682.2, 561.0, 436.6;^1^H − NMR (CD_3_OD, 100 MHz, δ in ppm) 8.28 (2H, s, CH), 7.60 (1H, s, CH), 7.58 (1H, d, *J* = 15.2 Hz, = CH), 7.06 (1H, d, *J* = 2.0 Hz, CH), 6.95 (1H, dd, *J* = 8.0, 2.0 Hz, CH), 6.78 (1H, d, *J* = 8.0 Hz, CH), 6.52 (1H, d, *J* = 15.2 Hz, = CH);^13^C − NMR (CD_3_OD, 100 MHz, δ in ppm) 167.84, 149.56, 146.95, 145.02, 142.54, 133.84 & 133.51 & 133.18 & 132.85 (q, *J* = 33.0 Hz, 2C), 128.92 & 126.22 & 123.52 &120.82 (q, *J* = 270.2 Hz, 2C), 128.05, 122.90, 120.46 & 120.42 (d, *J* = 3.5 Hz, 2C), 117.79, 117.54 & 117.51 & 117.47 & 117.43 & 117.39 (quint, *J* = 3.5 Hz), 116.64, 115.39; HRMS m/z of caffeic acid *N*-[3,5-bis(trifluoromethyl)phenyl] amide was observed at 392.0720 ([M + H]^+^, calculated for C_17_H_11_F_6_NO_3_H, 392.0716).

### Culturing of human keratinocyte (HaCaT) and DP cells

HaCaT and DP cells were cultivated in DMEM with 10% FBS, 1% l-glutamine, and 1% antibiotics in T75 flasks at 37 °C under a 5% CO_2_ atmosphere until 80% confluence before they were used for further experiments.

### Identification of steroid 5α-reductases in HaCaTs

Total RNA was extracted from HaCaT cells by using Purelink RNA mini purification kit (1IV11-12183-018A, Invitrogen, USA). The RNA was purified by DNase I treatment (Dnase I, Cat No. EN0521, Thermo Fisher Scientific, Rockford, IL, USA). cDNA was synthesized using RevertAid First Stand cDNA Synthesis Kit (Cat No. K1621 Thermo Fisher Scientific, Rockford, IL, USA). The specific forward and reverse primers for synthesis of cDNA was presented as following:

- Human Actin forward primer: 5′- ATGATGATATCGCCGCGCTC -3′

- Human Actin reverses primer: 5′- GCGCTCGGTGAGGATCTTCA -3′

- Human SRD5A1 forward primer: 5′- CTGATGCGAGGAGGAAAGCCTATGC-3′

- Human SRD5A1 reverses primer: 5′- GTCCAGATGCCTTTGCCTCACCTTG -3′

- Human SRD5A2 forward primer: 5′- TGCCTTCTGCACTGGAAATGGAGTC-3′

- Human SRD5A2 reverses primer: 5′- GGAGTGGGTTTGCTCTGGGTCTTTG -3′

### In vitro cytotoxic assay against human keratinocyte (HaCaT) and DP cells

The cytotoxicity of caffeic acid and its amide derivatives were evaluated with two cell lines, human keratinocyte (HaCaT) and DP cells, using the common cell viability assay with MTT. HaCaT and DP cells were seeded at 1 × 10^4^ cell/well in 96-well plates in a complete DMEM medium, incubated overnight, and treated with test compounds at 0–50 µM for 24 h. The stock solutions of all the test compounds were prepared in DMSO. Each compound was serially diluted in DMEM to achieve concentrations at 0.5–50 µM; DMEM was used as the negative control. After 24 h of treatment, the culture medium was removed, and cell viability was determined by adding 100 µL of 0.5 mg/mL MTT at 37 °C in darkness for 3 h. After removing the MTT solution, 100 µL of DMSO was added to each well to dissolve the purple formazan crystals. The absorbance was measured at 570 nm using a microplate reader, and the IC_50_ value was calculated. Each experiment was performed in triplicates.

### In vitro HaCaT-based SRD5A1 inhibitory assay

The HaCaT-based SRD5A1 inhibitory assay involves culturing the cells for 48 h, treating the cells with a compound for 24 h, and evaluating cell viability in parallel with the MTT assay and steroid 5α-reductase inhibitory activity assay using HPTLC. HaCaTs were seeded in 6-well plates at 2 × 10^5^ cells/well in DMEM with 0.1% DMSO and allowed to attach for 24 h. Then, 1 mL of 20 µM testosterone (T), the enzyme substrate, in DMEM with 0.1% DMSO was added. The controls were 1 mL of DMEM with 0.1% DMSO and 1 mL of 5.0 µM dutasteride (DU), the enzyme inhibitor. For the preliminary screening, caffeic acid (**1**) and two amides **2** and **3** were prepared at 40 µM, while amide **4** was prepared at 2 µM. For the evaluation of SRD5A1 inhibitory concentration, the *N*-[3,5-bis(trifluoromethyl)phenyl] amide of caffeic acid (**4**) was serially diluted and prepared at 0.4, 1.0, 2.0, 5.0, and 10.0 µM. Each test compound was dissolved in DMEM containing 0.1% DMSO. The cells were treated with 1 mL of each test compound at a non-toxic concentration. The cells were incubated in a total of 2 mL of the medium at 37 °C under a 5% CO_2_ atmosphere for 24 h. Therefore, the final concentration of test compounds was reduced by half. Afterward, the cell culture medium was separated from the cells, extracted with 2 mL of ethyl acetate, and centrifuged at 10,000 rpm at 25 °C for 5 min before a layer of 1 mL of ethyl acetate was collected. The extracted process was repeated twice by adding 0.5 mL ethyl acetate each time and collecting the layer of 0.5 mL of ethyl acetate. Next, 2 mL of the combining ethyl acetate extract was air-dried and reconstituted with 20 µL methanol. Then, 8 µL of the methanol solution was spotted on a silica gel 60 F254 glass HPTLC plate using a HPTLC sample loader (CAMAG Linomat 5). A mixture of cyclohexane: ethyl acetate: triethylamine solution (1.5:1:0.1 v/v) was used as the mobile phase. The HPTLC plate was developed twice, stained with 42.5% phosphoric acid in ethanol, and heated at 120 °C for 20 min. The product produced by 5α-reductase, dihydrotestosterone (DHT), was detected under UV light at 366 nm and captured by a TLC scanner CAMAG TLC Scanner 3. The obtaining chromatogram was visualized and analyzed by CAMAG HPTLC Software visionCATS. The inhibition of SRD5A1 was calculated according to the intensity of the DHT produced compared to that produced in the presence of the positive control, dutasteride (DU). In parallel, the viability of the attached treated cells was verified immediately after removing the cell culture medium for the MTT assay. Cell viability should be higher than 80% at each concentration of the test compound to avoid false-positive results. Each experiment was repeated in triplicates.

### Detection of mRNA level of SRD5A1 by reverse transcription quantitative real-time PCR (RT-qPCR)

HaCaT cells (2 × 10^5^ cells/well in 6 well-plates) were treated with compound **4** (0, 0.5, 1 and 2.5 µM) for 12 and 24 h. Then, total RNA was extracted by using Purelink RNA mini purification kit. The isolated RNA was purified by DNase I and further used as a template for synthesis of cDNA using RevertAid First Stand cDNA Synthesis Kit. Quantitative real-time PCR (RT-qPCR) was performed with a C100 Thermal Cycler (Bio-Rad CFX384 real-time pcr system) using Luna Universal qPCR Master Mix (M3003). Specific forward and reverse primers of the interested gene was indicated as following.

- Human GAPDH forward primer: 5′-GAGTCCACTGGCGTCTTCA-3′

- Human GAPDH reverses primer: 5′-TTCAGCTCAGGGATGACCTT-3′

- Human SRD5A1 forward primer: 5′-CGGGCATCGGTGCTTAATTT-3′

- Human SRD5A1 reverse primer: 5′-AAACGGGGATCTGTTACCCAG-3′

The reactions were incubated for 39 cycles of 95 °C for 5 s, 60 °C for 30 s and 65 °C for 5 s. The expression of target mRNA was determined as the relative comparison via the ΔCt method. The threshold (Ct) of target genes were normalized with GAPDH (Ct) values.

### Western blot analysis for detection of SRD5A1 protein expression

HaCaT cells (1 × 10^5^ cells/well in 6-well plates) were cultured in DMEM with or without compound **4** (0–2.5 µM) for 12 and 24 h. The cells were lysed with RIPA buffer containing 1% protease inhibitor and 1% phosphatase inhibitor on ice for 45 min. Then, the total protein from each sample was determined by bicinchoninic acid (BCA) protein assay kit (Cat No. 23225, Thermo Fisher Scientific, Rockford, IL, USA). The equal amount (40 µg) of protein sample was mixed with loading buffer and heated at 95 °C for 5 min before loaded to 10% sodium dodecyl sulfate–polyacrylamide gels (SDS-PAGE). The proteins were transferred onto PVDF membrane, which was further blocked with 5% BSA in TBST (25 mmol/L Tris–HCl; pH 7.4, 125 mmol/L NaCl, 0.1% Tween 20) at room temperature for 1 h. The primary antibody specific to SRD5A1 (1TFS-AB-PA575919, Thermo Fisher Scientific, Rockford, IL, USA) in dilution 1:1000 was added onto the membrane and incubated overnight at 4 °C. Then the membrane was washed 3 times × 7 min with TBST following with the immersion in the specific secondary antibody conjugated with horseradish peroxidase (HRP) for 1 h at room temperature. The signal of target protein was determined by using chemiluminescence HRP substrate (Thermo Scientific, Rockford, IL, USA) and the intensity of target protein was analyzed by analyst/PC densitometric software (Bio-Rad, Hercules, CA, USA).

### Whole cell-based kinetic studied of compound 4 with HaCaTs produced SRD5A1

The in vitro* whole* HaCaT cell-based assay for the kinetic inhibition against SRD5A1 included culturing the cells for 48 h, treating the cells with testosterone and compound **4** for 12 h and evaluating the % intensity of DHT and cell viability using HPTLC analysis and MTT assay, respectively. The HaCaT cells were seeded and incubated as same as the method shown in 3.8. For the evaluation of dose-dependent activity of testosterone, the different concentration of testosterone (0 − 50 μM) and DMEM with 0.1% DMSO were added. For the assessment of kinetic inhibition of compound **4** against HaCaT-produced SRD5A1, the cells were treated with various concentration of testosterone (0 − 50 μM) and compound **4** (0.2, 0.5, 1 and 2.5 μM). DMEM with 0.1% DMSO was used as negative control. Then, the cells were incubated for 12 h. The collection of cell culture medium, extraction process, sample preparation and HPTLC detection were prepared by the method mentioned above (3.8). The obtaining chromatogram was visualized by CAMAG HPTLC Software vision CATS and analyzed by Image J software. The percentage of DHT intensity was calculated based on the intensity of DHT normalized by the standard of DHT at 100 μM. At the same time, the cell viability of the treated cells was assessed by the MTT assay. Each experiment was repeated in triplicate.

### Statistical analysis

All experiments were performed independently three times and in triplicates; the data were presented as mean ± SD. Statistical analyses were carried out using GraphPad Prism 9.3.1 (San Diego, CA, USA). The differences between the means were analyzed using a one-way analysis of variance. A *p*-value < 0.05 was considered statistically significant.

### Molecular docking

The 3D structure of human SRD5A1 (P18405) was retrieved from the UniProtKB database which was derived from AlphaFold protein structure prediction^44^. The structure of compound **4,** finasteride, dutasteride, and the adducts of these compounds and NADP were built using Accelrys Discovery Studio 2.5 (Accelrys Software Inc, San Diego, CA, USA)^49^. The protonation state of 5α-reductase and the compounds was predicted by PDB2PQR^50^ and ChemAxon^51^, respectively. The binding mode of compound **4** and NADP-dihydro-**4** adduct inhibition compared with known drugs were studied using molecular docking with the GOLD program based on a genetic algorithm (GA)^52^.

The crystal structure of human SRD5A2 complexed with an NADP-dihydrofinasteride adduct (7BW1.pdb)^35^ was downloaded from Protein Data Bank. The 3D structure of both human SRD5A1 and SRD5A2 was superimposed. The NADP-dihydrofinasteride adduct was defined as the center of the active site for docking study. The 5α-reductase system was set as 6.5 Å for sphere docking and GOLD score for the scoring function. All systems were used as 100 independent docking poses and the structure with the highest GOLD score was selected to study. The GoldScore function combines force field terms with empirical terms to account for some of the deficiencies in pure force-field-based scoring functions^53^. It is optimized to predict the binding sites of the ligand considering factors such as the energy of hydrogen bonds, van der Waals (vdW) energy, metal interactions and torsion deformations, which is a molecular mechanics–like function with four terms as follows:

$$GOLD \, fitness \, = \, S_{hb(ext)} + \, S_{vdw(ext)} + S_{hb(int)} + \, S_{vdw(int)}$$

where *S*_*hb(ext)*_ is protein–ligand hydrogen-bond energy (external H-bond) and *S*_*vdw(ext)*_ is protein–ligand vdW energy (external vdW), *S*_*hb(int)*_ is the contribution of intramolecular hydrogen bonds in the ligand to Fitness and *S*_*vdw(int)*_ is the contribution due to intramolecular in the ligand. After the GOLD score and binding patterns were obtained, the mode of binding between the compounds and 5α-reductase was elucidated using Accelrys Discovery Studio 2.5 (Accelrys Software Inc, San Diego, CA, USA)^49^ and ChimeraX 1.1^54^.

### Molecular dynamics simulations

All-atom MD simulations of the SRD5A1 complexes were carried out using the AMBER20 under periodic boundary conditions. Both compound **4** and NADP-dihydro-**4** in complex with SRD5A1 were embedded in 1-oleoyl-2-palmitoyl-sn-glycero-3-phosphocholine (POPC) lipid bilayer followed by TIP3P water solvation and neutralization, using sodium and chloride ions at 150 mM as previously reported^35^. The geometries of the two ligands were optimized by HF/6-31(d) level of theory as per previously studies^55,56^ using the Gaussian09 program. The electrostatic potential (ESP) charges and restrained ESP (RESP) charges of compounds were obtained. The FF19SB and GAFF2 force fields were applied for protein and ligands, respectively. Any steric hindrance or improper geometry was removed by minimization using 5000 iterations of steepest descent (SD) followed by 10,000 iterations of conjugated gradient (CG). Then heated gradually to 310 K, using a Langevin thermostat was applied, while the Berendsen algorithm was used to regulate the pressure at 1 atm. The nonbonded interactions were considered using the short-range cutoff of 12 Å, whereas long-range electrostatic interactions were treated using Ewald’s method. Covalent bonds containing hydrogen atoms were constrained with the SHAKE algorithm. Finally, 1 μs NPT simulations at 310 K and 1 atom were performed for the production runs. Every 500 steps, the MD trajectories were collected for analysis. The RMSD, distance between the center of mass of actives site residues and compound, the number of contacts, intermolecular HB occupation, RMSF and B-factor were evaluated using the CPPTRAJ module. While the MM/(PB)GBSA and decomposition free energy were calculated by the MM/PBSA.py module. Noted that the distance and angle between the hydrogen bond donor (HD) and hydrogen acceptor (HA) were employed as a criterion for hydrogen bond calculations, with the distance and angle of ≤ 3.5 Å and ≥ 120°, respectively.

## Conclusions

In this study, a series of caffeic acid amide derivatives were designed and synthesized, with a focus on the addition of amide substituents that were similar to finasteride and dutasteride. HaCaTs produced SRD5A1 as a major isozyme, which facilitates the use of a HaCaT cell-based assay for the screening of SRD5A1 inhibitor for androgenic alopecia. Evaluation of the inhibitory activity against SRD5A1 using a HaCaT-based assay with direct DHT detection by HPTLC analysis revealed a caffeic acid *N*-[3,5-bis(trifluoromethyl)phenyl] amide derivative (compound **4**) as a potential non-steroidal SRD5A1 suppressor, with an inhibitory activity at IC_50_ 1.44 ± 0.13 µM and a low cytotoxicity at IC_50_ 29.99 ± 8.69 µM. Compound **4** displayed three-fold more potent inhibitory activity against SRD5A1 than avicequinone C, a previously reported natural non-steroidal inhibitor. Importantly, compound **4** at a concentration of 2.5 µM regulated the activity of SRD5A1 via suppression of SRD5A1 protein expression and potentially directed SRD5A1 inhibition via a mixed mode of inhibition. The underlying inhibitory mechanism of compound **4** was also investigated by conducting a whole-cell kinetic study, which gave a K_i_ value of 2.382 µM under specific conditions. The stability of SRD5A1 is crucial to limit the in vitro binding study. Thus, the direct SRD5A1 inhibition behavior of compound **4** was predicted by MD simulations. Furthermore, the binding interactions of compound **4** with human SRD5A1 generated by AlphaFold were studied via molecular docking and molecular dynamics simulations. The NADP-dihydro-**4** adduct stabilized within the catalytic pocket site of SRD5A1 by hydrophobically interacting with the key M119 residue along with the increased hydrogen bond interactions that were stronger than those of finasteride and dutasteride. The in vitro and in silico results indicate that compound **4** modulates SRD5A1 function, leading to a lower level of DHT production and SRD5A1 protein suppression. Therefore, compound **4** is a promising non-steroidal SRD5A1 suppressor and should be further investigated in the clinical development stage for treating androgenic alopecia.

## Electronic supplementary material

Below is the link to the electronic supplementary material.

Supplementary Information 1.

Supplementary Information 2.

Supplementary Information 3.

Supplementary Information 4.

Supplementary Information 5.

Supplementary Video 1.

Supplementary Video 2.

Supplementary Information 6.

## Data Availability

The data described and analyzed in this study are available from the corresponding author upon reasonable request.

## References

[1] Azzouni F, Godoy A, Li Y, Mohler J (2012). The 5α-reductase isozyme family: A review of basic biology and their role in human diseases. Adv. Urol..

[2] Randall VA (1994). Role of 5α-reductase in health and disease. Baillieres Clin. Endocrinol. Metab..

[3] Godoy A (2011). 5α-reductase type 3 expression in human benign and malignant tissues: A comparative analysis during prostate cancer progression. Prostate.

[4] Jain R, De-Eknamkul W (2014). Potential targets in the discovery of new hair growth promoters for androgenic alopecia. Expert Opin. Ther. Targets.

[5] Sawaya ME, Price VH (1997). Different levels of 5α-reductase type I and II, aromatase, and androgen receptor in hair follicles of women and men with androgenetic alopecia. J. Investig. Dermatol..

[6] Varothai S, Bergfeld WF (2014). Androgenetic alopecia: An evidence-based treatment update. Am. J. Clin. Dermatol..

[7] Zhou Z (2019). The efficacy and safety of dutasteride compared with finasteride in treating men with androgenetic alopecia: A systematic review and meta-analysis. Clin. Interv. Aging.

[8] Starace M, Orlando G, Alessandrini A, Piraccini BM (2020). Female androgenetic alopecia: An update on diagnosis and management. Am. J. Clin. Dermatol..

[9] Dhurat R (2020). 5-Alpha reductase inhibitors in androgenetic alopecia: Shifting paradigms, current concepts, comparative efficacy, and safety. Dermatol. Ther..

[10] Trilisnawati D (2021). Update treatment of male androgenetic alopecia. Period. Dermatol. Venereol..

[11] Holt DA (1990). Inhibition of steroid 5 alpha-reductase by unsaturated 3-carboxy steroids. J. Med. Chem..

[12] Occhiato EG, Guarna A, Danza G, Serio M (2004). Selective non-steroidal inhibitors of 5α-reductase type 1. J. Steroid Biochem. Mol. Biol..

[13] Pérez-Ornelas V (2005). New 5α-reductase inhibitors. In vitro and in vivo effects. Steroids.

[14] Salem OI (2006). Novel 5α-reductase inhibitors: Synthesis, structure-activity studies, and pharmacokinetic profile of phenoxybenzoylphenyl acetic acids. J. Med. Chem..

[15] Aggarwal S, Thareja S, Verma A, Bhardwaj TR, Kumar M (2010). An overview on 5α-reductase inhibitors. Steroids.

[16] Kim S, Kim Y-U, Ma E (2012). Synthesis and 5α-reductase inhibitory activity of C21 steroids having 1, 4-diene or 4, 6-diene 20-ones and 4-azasteroid 20-oximes. Molecules.

[17] Karnsomwan W, Netcharoensirisuk P, Rungrotmongkol T, De-Eknamkul W, Chamni S (2017). Synthesis, biological evaluation and molecular docking of avicequinone C analogues as potential steroid 5α-reductase inhibitors. Chem. Pharm. Bull..

[18] Seo E-K (2002). Inhibitors of 5α-reductase type I in LNCaP cells from the roots of angelica koreana. Planta Med..

[19] Lao Z (2021). Physcion, A novel inhibitor of 5α-reductase that promotes hair growth in vitro and in vivo. Arch. Dermatol. Res..

[20] Hirata N, Tokunaga M, Naruto S, Iinuma M, Matsuda H (2007). Testosterone 5α-reductase inhibitory active constituents of piper nigrum leaf. Biol. Pharm. Bull..

[21] Srivilai J (2017). Anti-androgenic curcumin analogues as steroid 5α-reductase inhibitors. Med. Chem. Res..

[22] Lee H-H, Ho C-T, Lin J-K (2004). Theaflavin-3, 3′-digallate and penta-O-galloyl-β-D-glucose inhibit rat liver microsomal 5α-reductase activity and the expression of androgen receptor in LNCaP prostate cancer cells. Carcinogenesis.

[23] Murata K (2012). Inhibitory activities of puerariae flos against testosterone 5α-reductase and its hair growth promotion activities. J. Nat. Med..

[24] Jain R, Monthakantirat O, Tengamnuay P, De-Eknamkul W (2014). Avicequinone C isolated from avicennia marina exhibits 5α-reductase-type 1 inhibitory activity using an androgenic alopecia relevant cell-based assay system. Molecules.

[25] Jain R, Monthakantirat O, Tengamnuay P, De-Eknamkul W (2015). Identification of a new plant extract for androgenic alopecia treatment using a non-radioactive human hair dermal papilla cell-based assay. BMC Complement. Altern. Med..

[26] Spagnol CM (2019). In vitro methods to determine the antioxidant activity of caffeic acid. Spectrochim. Acta Part A Mol. Biomol. Spectrosc..

[27] Da-Cunha FM (2004). Caffeic acid derivatives: In vitro and in vivo anti-inflammatory properties. Free Radic. Res..

[28] Genaro-Mattos TC, Maurício ÂQ, Rettori D, Alonso A, Hermes-Lima M (2015). Antioxidant activity of caffeic acid against iron-induced free radical generation—a chemical approach. PLoS ONE.

[29] Agunloye OM (2019). Cardio-protective and antioxidant properties of caffeic acid and chlorogenic acid: Mechanistic role of angiotensin converting enzyme, cholinesterase and arginase activities in cyclosporine induced hypertensive rats. Biomed. Pharmacother..

[30] Mohammed FZ, Al-Hussaini ASE-D, El-Shehabi ME-S (2015). Antidiabetic activity of caffeic acid and 18î’-glycyrrhetinic acid and its relationship with the antioxidant property. Asian J. Pharm. Clin. Res..

[31] Liu Z, Fu J, Shan L, Sun Q, Zhang W (2014). Synthesis, preliminary bioevaluation and computational analysis of caffeic acid analogues. Int. J. Mol. Sci..

[32] Miyata S, Oda Y, Matsuo C, Kumura H, Kobayashi K (2014). Stimulatory effect of brazilian propolis on hair growth through proliferation of keratinocytes in mice. J. Agric. Food Chem..

[33] Opella SJ (2013). Structure determination of membrane proteins by nuclear magnetic resonance spectroscopy. Annu. Rev. Anal. Chem..

[34] Han Y (2021). Crystal structure of steroid reductase SRD5A reveals conserved steroid reduction mechanism. Nat. Commun..

[35] Xiao Q (2020). Structure of human steroid 5α-reductase 2 with the anti-androgen drug finasteride. Nat. Commun..

[36] Bull HG (1996). Mechanism-based inhibition of human steroid 5α-reductase by finasteride: Enzyme-catalyzed formation of NADP−dihydrofinasteride, a potent bisubstrate analog inhibitor. J. Am. Chem. Soc..

[37] Koseki J (2015). Inhibition of rat 5α-reductase activity and testosterone-induced sebum synthesis in hamster sebocytes by an extract of Quercus acutissima cortex. Evid.-Based Complem. Altern. Med..

[38] Russell DW, Wilson JD (1994). Steroid 5α-reductase: Two genes/two enzymes. Annu. Rev. Biochem..

[39] Srivilai J, Minale G, Scholfield CN, Ingkaninan K (2019). Discovery of natural steroid 5 alpha-reductase inhibitors. Assay Drug Dev. Technol..

[40] Shen SH, Wertz DL, Klinman JP (2012). Implication for functions of the ectopic adipocyte copper amine oxidase (AOC3) from purified enzyme and cell-based kinetic studies. PLoS ONE.

[41] Li X, Chen C, Singh SM, Labire F (1995). The enzyme and inhibitors of 4-ene-3-oxosteroid 5α-oxidoreductase. Steroids.

[42] Dhingra N (2021). Steroidal 5α-reductase: A therapeutic target for prostate disorders. Oxidoreductase.

[43] Silverstein TP (2019). When both K(m) and V(max) are altered, Is the enzyme inhibited or activated?. Biochem. Mol. Biol. Educ..

[44] Varadi M (2022). AlphaFold protein structure database: Massively expanding the structural coverage of protein-sequence space with high-accuracy models. Nucleic Acids Res..

[45] Huang SY, Zou X (2007). Efficient molecular docking of NMR structures: Application to HIV-1 protease. Protein Sci..

[46] Frye SV (1995). Structure-activity relationships for inhibition of type 1 and 2 human 5 alpha-reductase and human adrenal 3 beta-hydroxy-delta 5-steroid dehydrogenase/3-keto-delta 5-steroid isomerase by 6-azaandrost-4-en-3-ones: Optimization of the C17 substituent. J. Med. Chem..

[47] Holt DA (1995). Benzophenone- and indolecarboxylic acids: Potent type-2 specific inhibitors of human steroid 5 alpha-reductase. J. Med. Chem..

[48] Pettersen EF (2004). UCSF Chimera–a visualization system for exploratory research and analysis. J. Comput. Chem..

[49] Wu GS, Robertson DH, Brooks CL, Vieth M (2003). Detailed analysis of grid-based molecular docking: A case study of CDOCKER—a CHARMm-based MD docking algorithm. J. Comput. Chem..

[50] Dolinsky TJ (2007). PDB2PQR: Expanding and upgrading automated preparation of biomolecular structures for molecular simulations. Nucleic Acids Res..

[51] Marvin was used for drawing, displaying and characterizing chemical structures, substructures and reactions, marvin 17.21.0, ChemAxon. https://www.chemaxon.com (2022).

[52] Jones G, Willett P, Glen RC, Leach AR, Taylor R (1997). Development and validation of a genetic algorithm for flexible docking. J. Mol. Biol..

[53] Annamala MK, Inampudi KK, Guruprasad L (2007). Docking of phosphonate and trehalose analog inhibitors into *M. tuberculosis* mycolyltransferase Ag85C: Comparison of the two scoring fitness functions GoldScore and ChemScore, in the GOLD software. Bioinformation.

[54] Pettersen EF (2021). UCSF ChimeraX: Structure visualization for researchers, educators, and developers. Protein Sci..

[55] Sanachai K (2020). Insights into the binding recognition and susceptibility of tofacitinib toward janus kinases. ACS Omega.

[56] Sanachai K, Mahalapbutr P, Sanghiran Lee V, Rungrotmongkol T, Hannongbua S (2021). In silico elucidation of potent inhibitors and rational drug design against SARS-CoV-2 papain-like protease. J. Phys. Chem. B.

