# Supplementary material for: Caffeic acid N-[3,5-bis(trifluoromethyl)phenyl] amide as a non-steroidal inhibitor for steroid 5α-reductase type 1 using a human keratinocyte cell-based assay and molecular dynamics
Source: Sci Rep. 2022 Dec 2;12:20858. doi: 10.1038/s41598-022-25335-7 (PMC9718795; doi:10.1038/s41598-022-25335-7)
Supplement: Supplementary file 5 — Supplementary Information 5. [file 41598_2022_25335_MOESM5_ESM.docx]

**Supporting Information**

**Caffeic Acid *N*-[3,5-Bis(trifluoromethyl)phenyl] Amide as** **a** **Non-Steroidal Inhibitor for Steroid 5α-Reductase Type 1 Using a Human Keratinocyte Cell-Based Assay and Molecular Dynamics**

**Aye Chan Khine Lin^1,2,3^, Ponsawan Netcharoensirisuk^2,4^, Kamonpan Sanachai^5^, Warongrit Sukma^1,2,3^, Chaisak Chansriniyom^2,3^, Chatchai Chaotham^6,7^, Wanchai De-Eknamkul^2,4^, Thanyada Rungrotmongkol^8,9^, Supakarn Chamni^1,2,3,^***

^1^ Pharmaceutical Sciences and Technology Program, Faculty of Pharmaceutical Sciences, Chulalongkorn University, Bangkok 10330, Thailand

^2^ Department of Pharmacognosy and Pharmaceutical Botany, Faculty of Pharmaceutical Sciences, Chulalongkorn University, Bangkok 10330, Thailand

^3^ Natural Products and Nanoparticles Research Unit (NP2), Chulalongkorn University, Bangkok 10330, Thailand; supakarn.c@pharm.chula.ac.th

^4^ Natural Product Biotechnology Research Unit, Chulalongkorn University, Bangkok 10330, Thailand; Wanchai.D@chula.ac.th

^5^ Department of Biochemistry, Faculty of Science, Khon Kaen University, Khon Kaen 40002, Thailand; kamosa@kku.ac.th

^6^ Department of Biochemistry and Microbiology, Faculty of Pharmaceutical Sciences, Chulalongkorn University, Bangkok 10330, Thailand

^7^ Center of Excellence in Cancer Cell and Molecular Biology, Faculty of Pharmaceutical Sciences, Chulalongkorn University, Bangkok 10330, Thailand; Chatchai.C@chula.ac.th

^8^ Center of Excellence in Structural and Computational Biology, Department of Biochemistry, Faculty of Science, Chulalongkorn University, Bangkok 10330; t.rungrotmongkol@gmail.com

^9^ Program in Bioinformatics and Computational Biology, Graduate School, Chulalongkorn University, Bangkok 10330, Thailand

* Corresponding author: supakarn.c@pharm.chula.ac.th; Tel.: +662-218-8357

| **Table of Content** | **Page** |
| --- | --- |
| **Figure S1** Preliminary screening of SRD5A1 inhibitory activity of caffeic acid (**1**) and amide derivatives **2−4** | S4 |
| **Figure S2** Full-length HPTLC chromatogram of caffeic acid *N*-[3,5-bis(trifluoro methyl)phenyl] amide (**4**) toward steroid 5α-reductase inhibitory activity using a HaCaT-based assay | S5 |
| **Figure S3** ^1^H−NMR spectrum of **2** in CD_3_OD | S6 |
| **Figure S4** ^13^C−NMR spectrum of **2** in CD_3_OD | S6 |
| **Figure S5** ^1^H−NMR spectrum of **3** in CD_3_OD | S7 |
| **Figure S6** ^13^C−NMR spectrum of **3** in CD_3_OD | S7 |
| **Figure S7** ^1^H−NMR spectrum of **4** in CD_3_OD | S8 |
| **Figure S8** ^13^C−NMR spectrum of **4** in CD_3_OD | S8 |
| **Figure S9** Original Western blot for detection of SRD5A1 protein expression at 12 h | S9 |
| **Figure S10** Original Western blot for detection of SRD5A1 protein expression at 24 h | S9 |
|  |  |
| **Table of Content** | **Page** |
| **Figure S11** HPTLC chromatogram for the evaluation of DHT intensity by SRD5A1 enzyme-based assay using crude enzyme extracted from HaCaTs | S10 |
| **Figure S12** HPTLC chromatogram for the evaluation of DHT intensity by SRD5A1 enzyme-based assay using purified enzyme extracted from HaCaTs | S10 |
| **Figure S13** HPTLC chromatogram for the HaCaT cell based kinetic study of compound **4** against SRD5A1 with various testosterone concentrations without inhibitor | S11 |
| **Figure S14** HPTLC chromatogram for the HaCaT cell based kinetic study of compound **4** against SRD5A1 with compound **4** (2.5 μM) and testosterone (10 μM) at 6 h. | S11 |
| **Figure S15** HPTLC chromatogram for the HaCaT cell based kinetic study of compound **4** against SRD5A1 with compound **4** (2.5 μM) and testosterone (10 μM) at 12 h. | S12 |
| **Figure S16** HPTLC chromatogram for the HaCaT cell based kinetic study of compound **4** against SRD5A1 with compound **4** (2.5 μM) and testosterone (10 μM) at 24 h. | S12 |
| **Figure S17** HPTLC chromatogram for the HaCaT cell based kinetic study of compound **4** against SRD5A1 with various testosterone concentrations and compound **4** (0.2 μM) | S13 |
| **Figure S18** HPTLC chromatogram for the HaCaT cell based kinetic study of compound **4** against SRD5A1 with various testosterone concentrations and compound **4** (0.5 μM) | S13 |
| **Figure S19** HPTLC chromatogram for the HaCaT cell based kinetic study of compound **4** against SRD5A1 with various testosterone concentrations and compound **4** (1.0 μM) | S14 |
| **Figure S20** HPTLC chromatogram for the HaCaT cell based kinetic study of compound **4** against SRD5A1 with various testosterone concentrations and compound **4** (2.5 μM) | S14 |
| **Figure S21** Sequence alignment between human SRD5A1 and SRD5A2. The red box represents the catalytic residues of SRD5A1 (E60, Y95 and M119) and SRD5A2 (E57, Y91 and R114) | S15 |
| **Figure S22** Superimposition of NADP-dihydrofinasteride adduct against SRD5A1 and SRD5A2 the redocking pose (black) with the crystal structure of ligand (green) | S15 |
| **Figure S23** 2D structure of NADP-dihydro-**4** | S16 |
| **Figure S24** Snapshot from the molecular dynamics (MD) trajectory derived from last 100 ns simulation (Run 1) of compound **4** and and its adduct NADP-dihydro-**4** against SRD5A1 | S16 |
| **Figure S25** The plots of #H-bonds of compound **4** (Runs 1 and 2) and NADP**-**dihydro**-4** (Run 1) in complex with SRD5A1 along with the 1**-**μs MD simulations. The angle between the hydrogen bond donor (HD) and hydrogen acceptor (HA) were employed as a criterion for strong hydrogen bond calculations of NADP**-**dihydro**-4** system, with the distance and angle of ≤ 3.5 Å and ≥ 120^o^, respectively. | S17 |
| **Figure S26** The plots of #H-bonds of compound **4** and NADP**-**dihydro**-4** in complex with SRD5A1 along with the 0.7**-**μs (Run 3) and 0.5**-**μs (Runs 2 and 3) MD simulations, respectively. The angle between the hydrogen bond donor (HD) and hydrogen acceptor (HA) were employed as a criterion for strong hydrogen bond calculations of NADP**-**dihydro**-4** system, with the distance and angle of ≤ 3.5 Å and ≥ 120^o^, respectively. | S17 |
|  |  |
| **Table of Content** | **Page** |
| **Figure S27** The plots of the RMSD of compound **4** and NADP**-**dihydro**-4**, the distance measured between the centers of mass of compound 4 and NADP-dihydro**-**4 and binding residues (d_L-Binding site_), # Atom contacts and #H-bonds of compound **4** and NADP**-**dihydro**-4** in complex with SRD5A1 along with the 0.7**-**μs (Run 3) and 0.5**-**μs (Runs 2 and 3) MD simulations, respectively. The angle between the hydrogen bond donor (HD) and hydrogen acceptor (HA) were employed as a criterion for strong hydrogen bond calculations of NADP-dihydro**-4** system, with the distance and angle of ≤ 2.8 Å and ≥ 150^o^, respectively. | S18 |
| **Table S1** Dihydrotestosterone (DHT) formation from the HaCaT cell based kinetic study with various testosterone (substrate) concentrations. | S19 |
| **Table S2** Dihydrotestosterone (DHT) formation from the HaCaT cell based kinetic study with various testosterone (substrate) concentrations and various compound **4** concentrations. | S19 |
| **Table S3** Lipinski’s rule of five prediction of caffeic acid, its amide derivatives and  steroid 5α-reductase inhibited drugs | S19 |
| **Video S1** MD simulation of compound **4/**SRD5A1 complex derived from the last 100 ns of the Run 1 simulation  **Video S2** MD simulation of NADP-dihydro-**4**/SRD5A1 complex derived from the last 100 ns of the Run 1 simulation  **File S1** The mdcrd file of compound **4/**SRD5A1 complex derived from the last 100 ns of the Run 1 simulation.  **File S2** The prmtop file of compound **4/**SRD5A1 complex derived from the last 100 ns of the Run 1 simulation.  **File S3** The mdcrd file of NADP-dihydro-**4/**SRD5A1 complex derived from the last 100 ns of the Run 1 simulation.  **File S4** The prmtop file of NADP-dihydro-**4/**SRD5A1 complex derived from the last 100 ns of the Run 1 simulation. | S20  S20  S20  S20  S20  S20 |


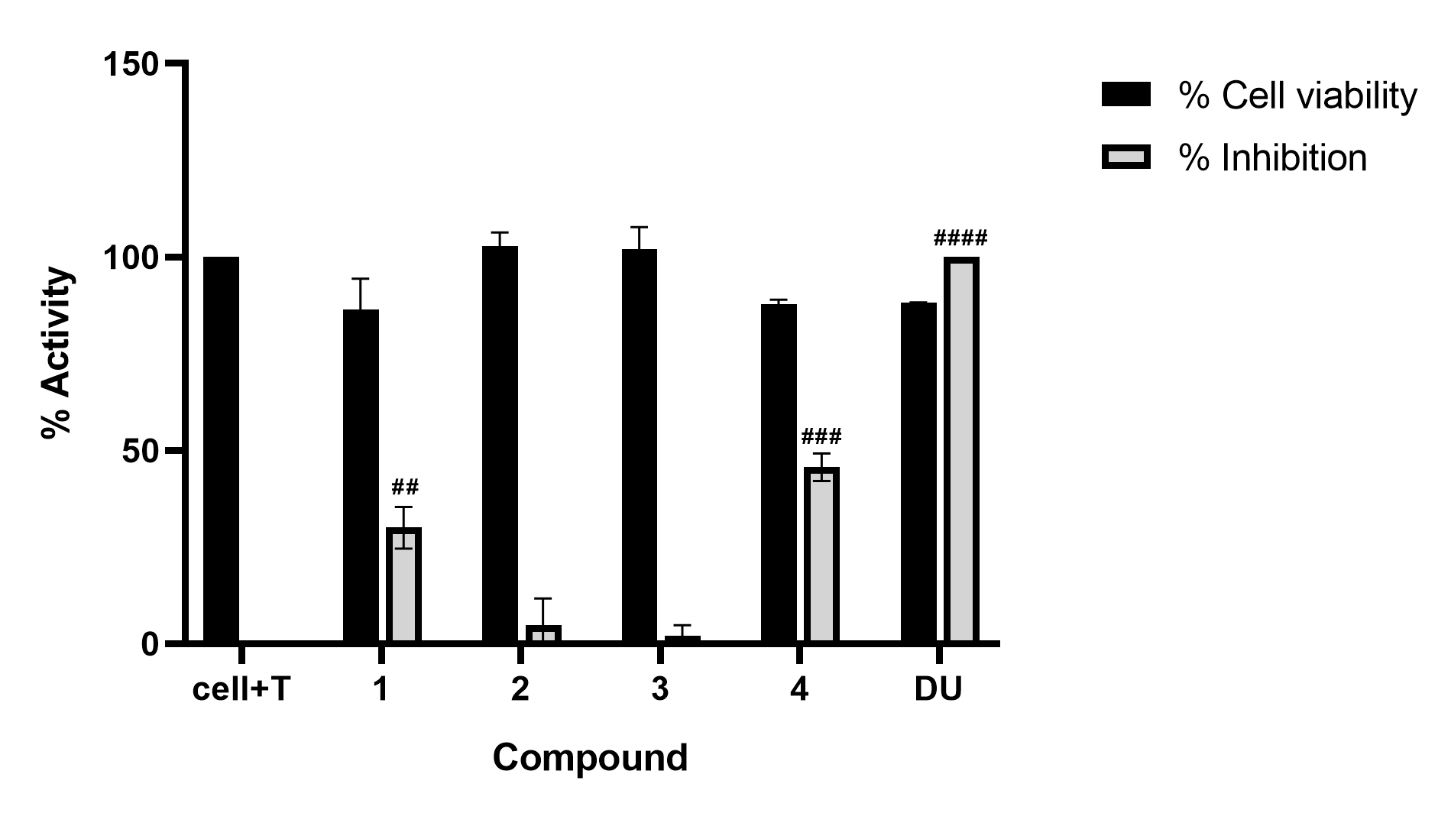


**Figure S1** Preliminary screening of SRD5A1 inhibitory activity of caffeic acid (**1**) and amide derivatives **2−4**. Data of inhibition display ##*p* < 0.05, ###*p* < 0.005 and ####*p* < 0.0001 versus non-treated control.

Caffeic acid (**1**), its amide derivatives **2** and **3** were evaluated at the concentrations of 20 μM and compound **4** was evaluated at 1 μM.


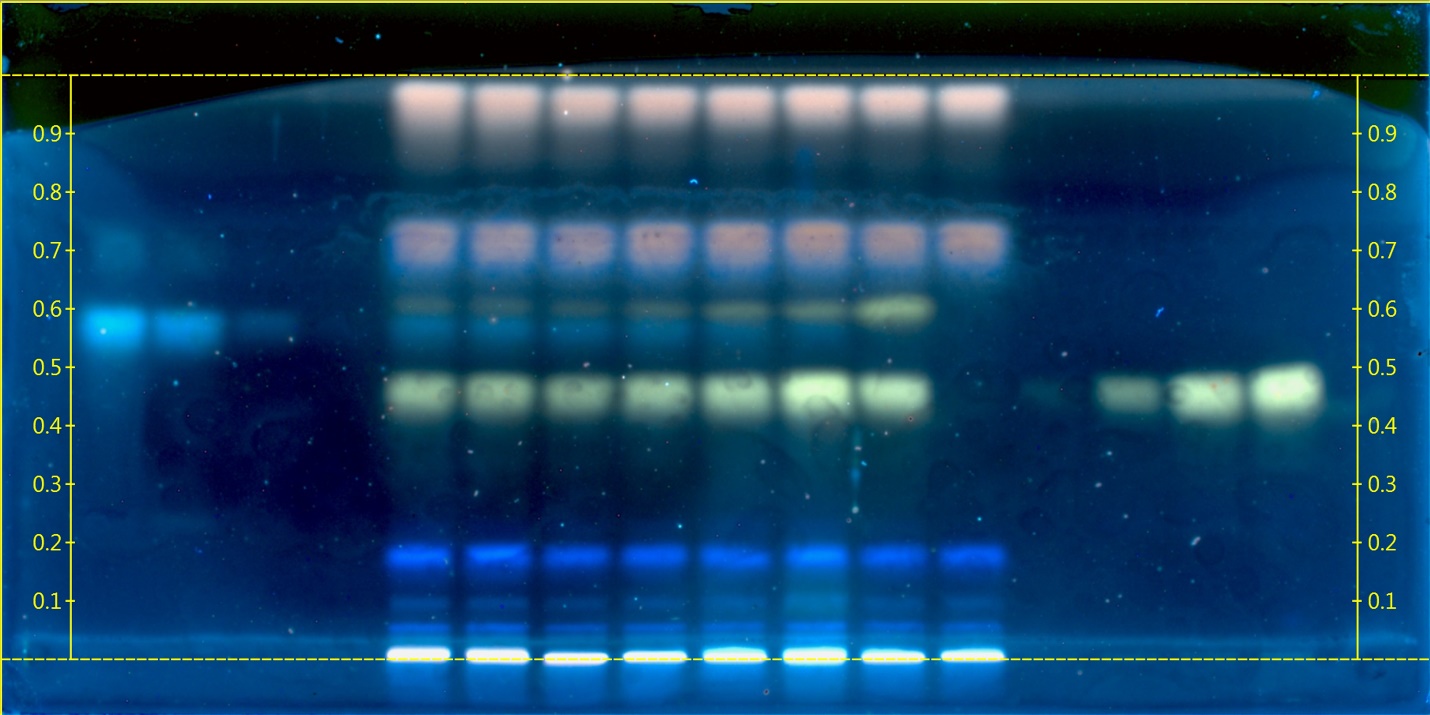


**Figure S2** Full-length and no exposure adjustment HPTLC chromatogram of caffeic acid *N*-[3,5-bis(trifluoro methyl)phenyl] amide (**4**) toward steroid 5α-reductase inhibitory activity using a HaCaT-based assay. (a) HPTLC chromatogram. Lanes 1−4, dihydrotestosterone (DHT) standards at 1000, 500, 100, and 10 μM. Lane 5, HaCaTs (Cell) treated with 10 μM testosterone (T). Lanes 6−10, HaCaTs treated with compound **4** at 0.2, 0.5, 1.0, 2.5, and 5.0 μM. Lane 11, HaCaTs treated with 2.5 μM dutasteride (DU). Lane 12, Cell refers to HaCaTs. Lanes 12−16, testosterone (T) standards at 10, 100, 500, and 1000 μM. The obtaining chromatogram was visualized by CAMAG HPTLC Software vision CATS.


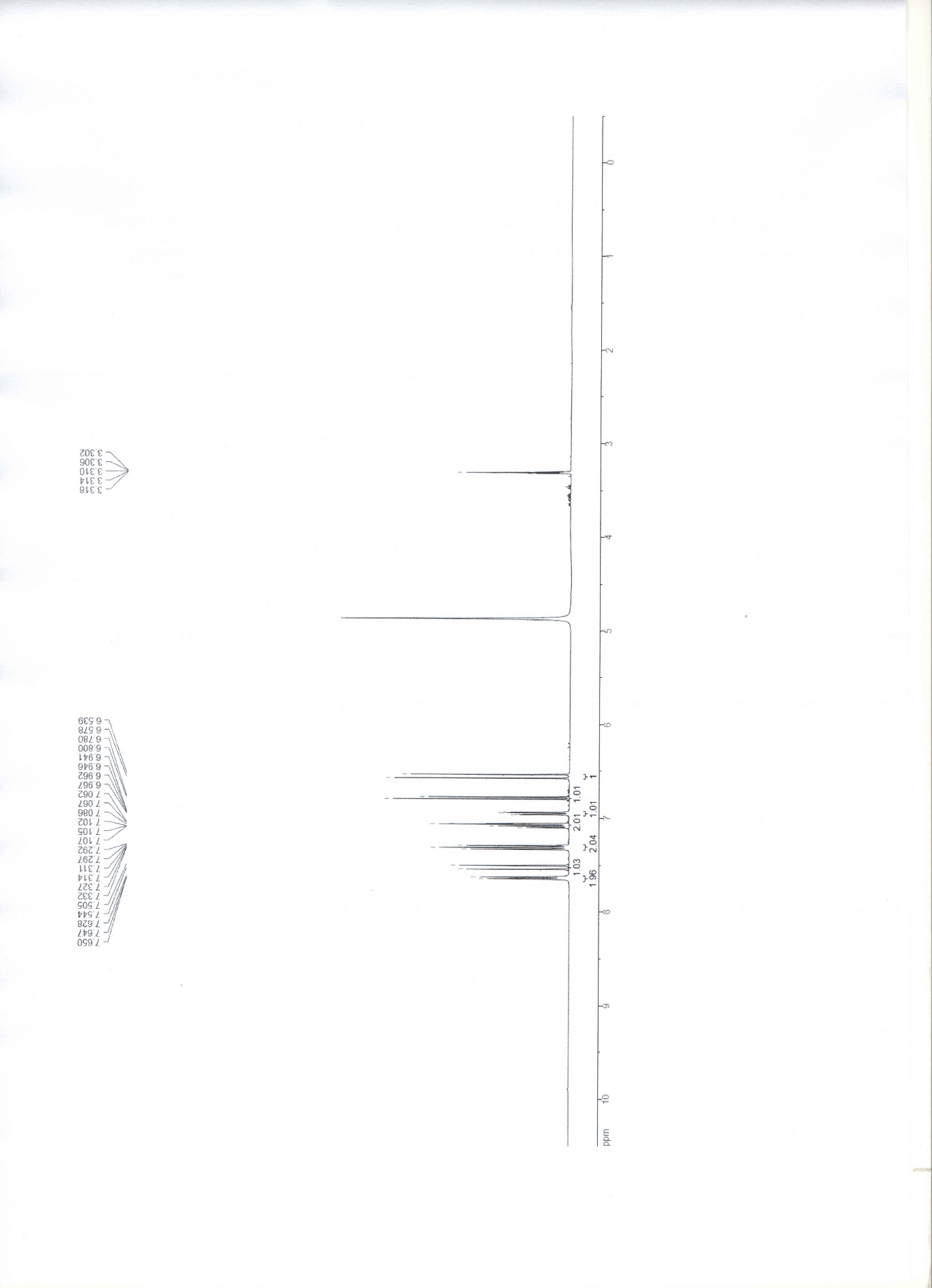


**Figure S3** ^1^H−NMR spectrum of **2** in CD_3_OD


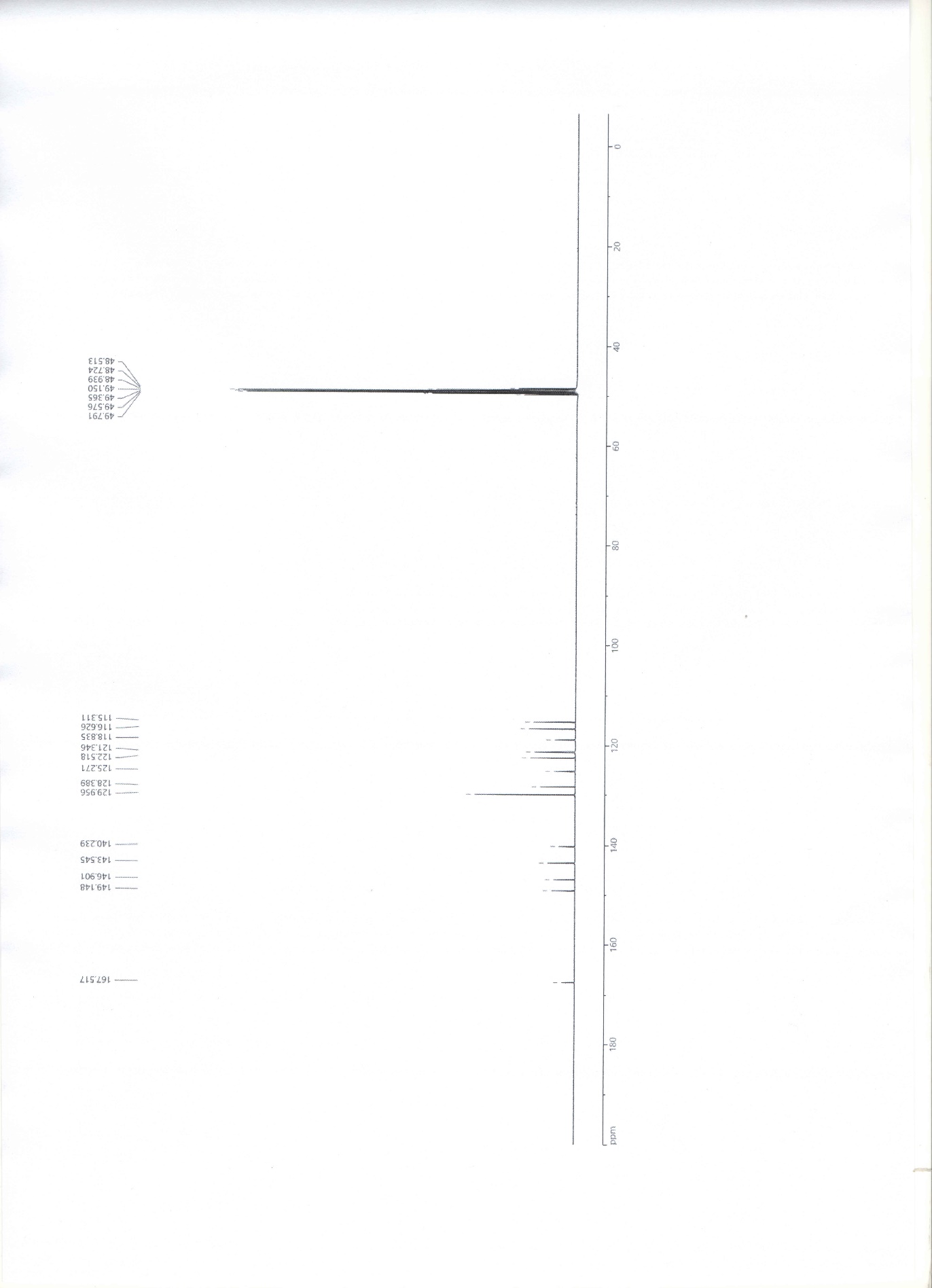


**Figure S4** ^13^C−NMR spectrum of **2** in CD_3_OD


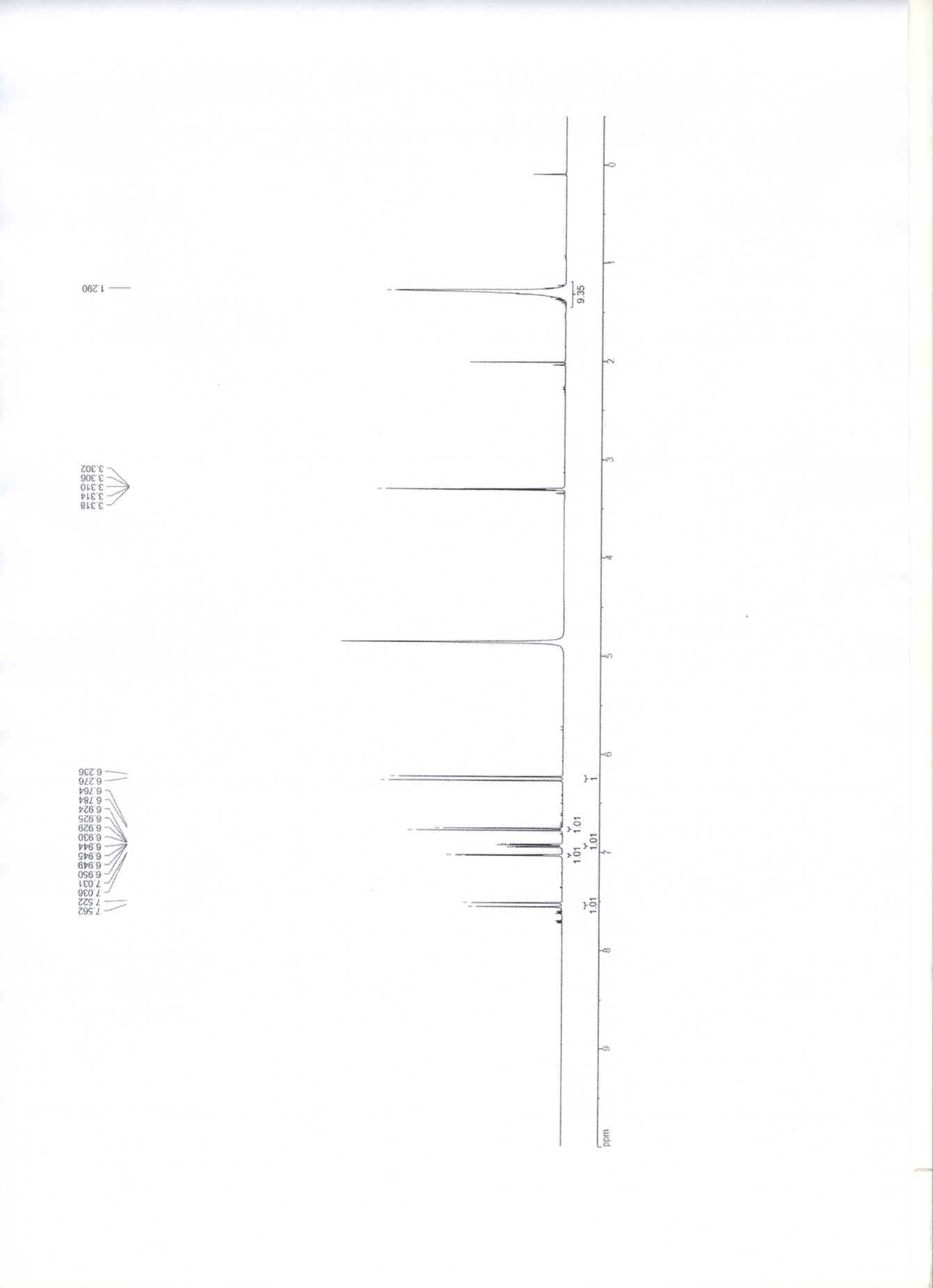


**Figure S5** ^1^H−NMR spectrum of **3** in CD_3_OD


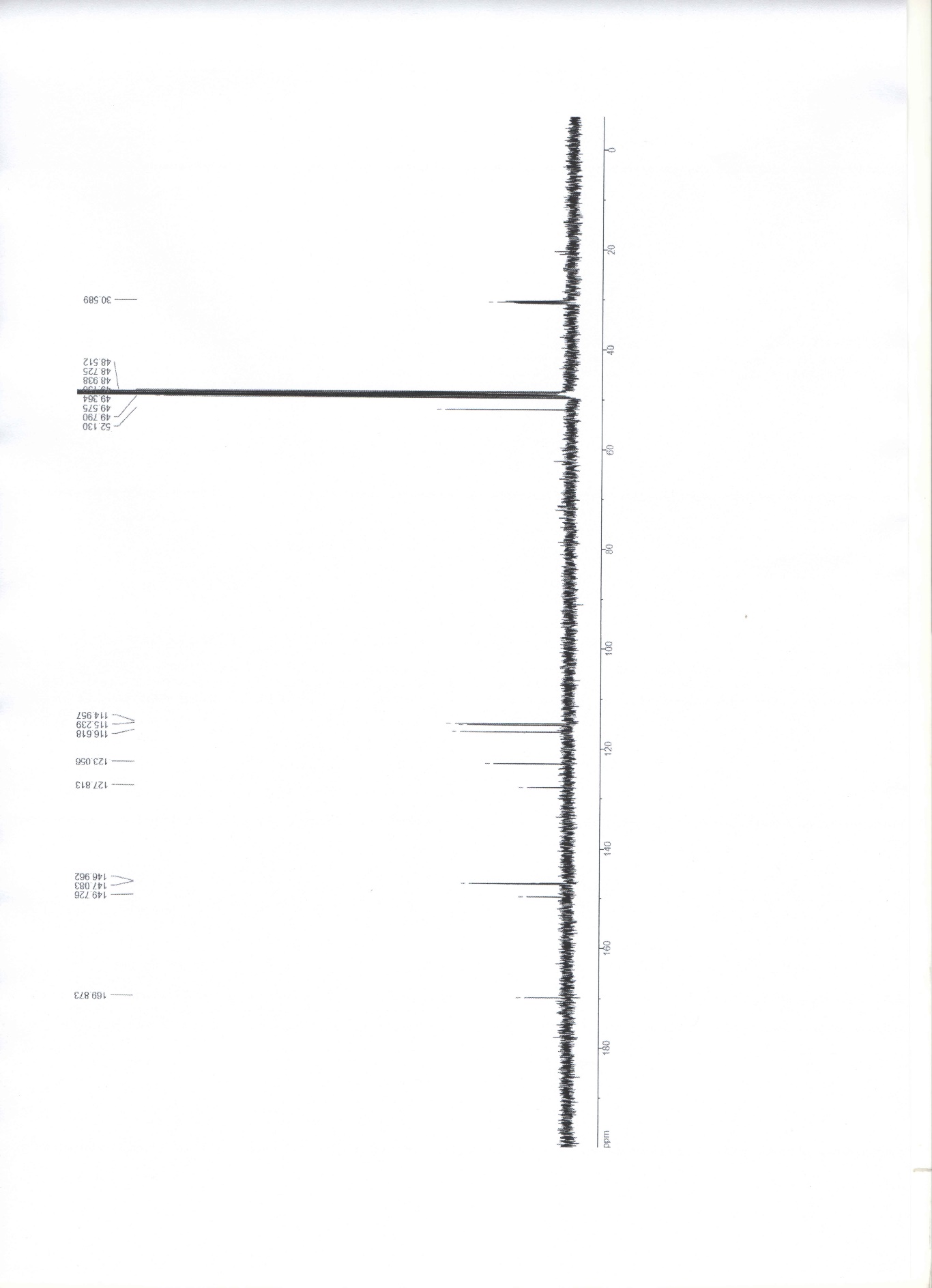


**Figure S6** ^13^C−NMR spectrum of **3** in CD_3_OD


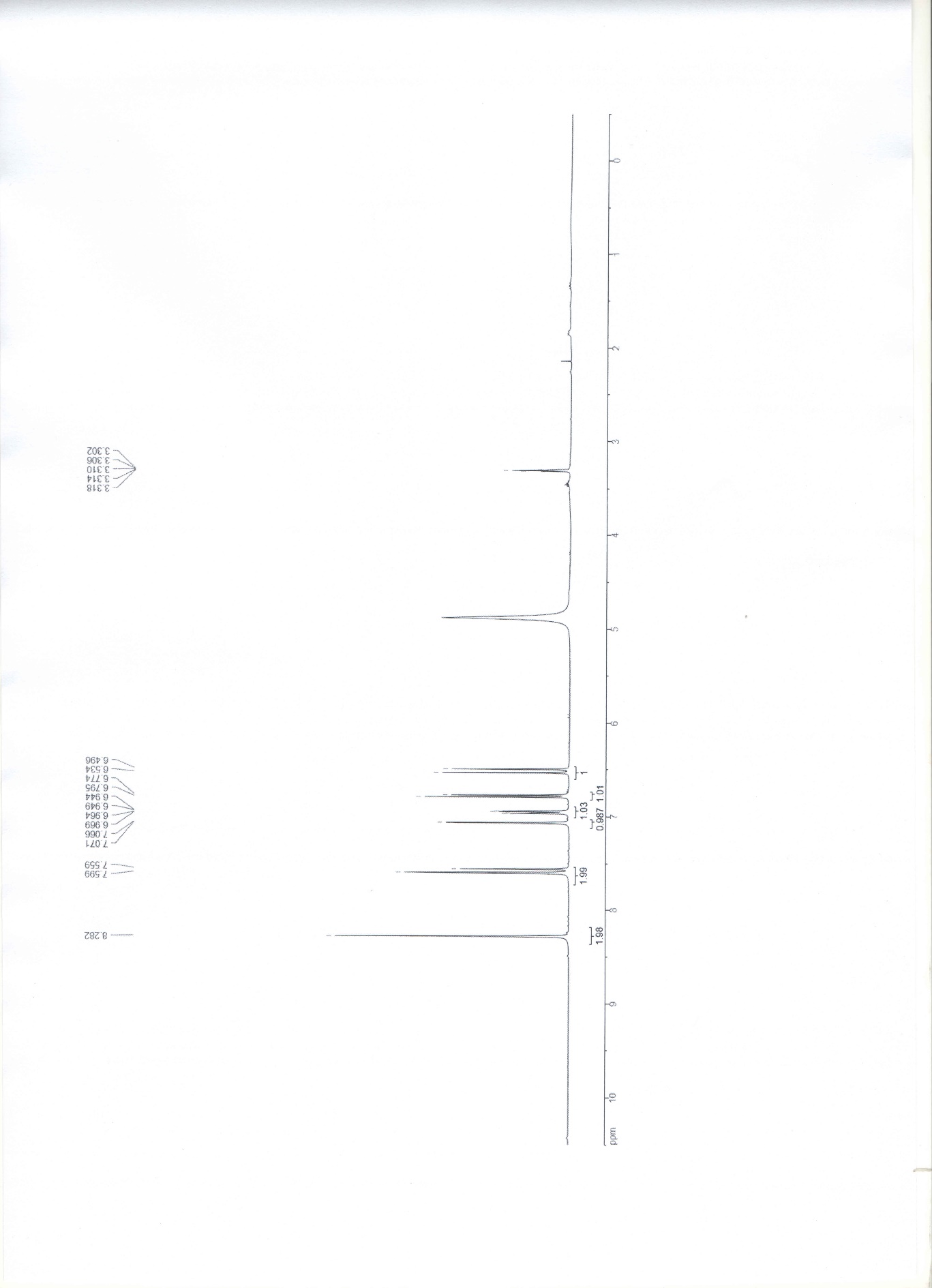


**Figure S7** ^1^H−NMR spectrum of **4** in CD_3_OD


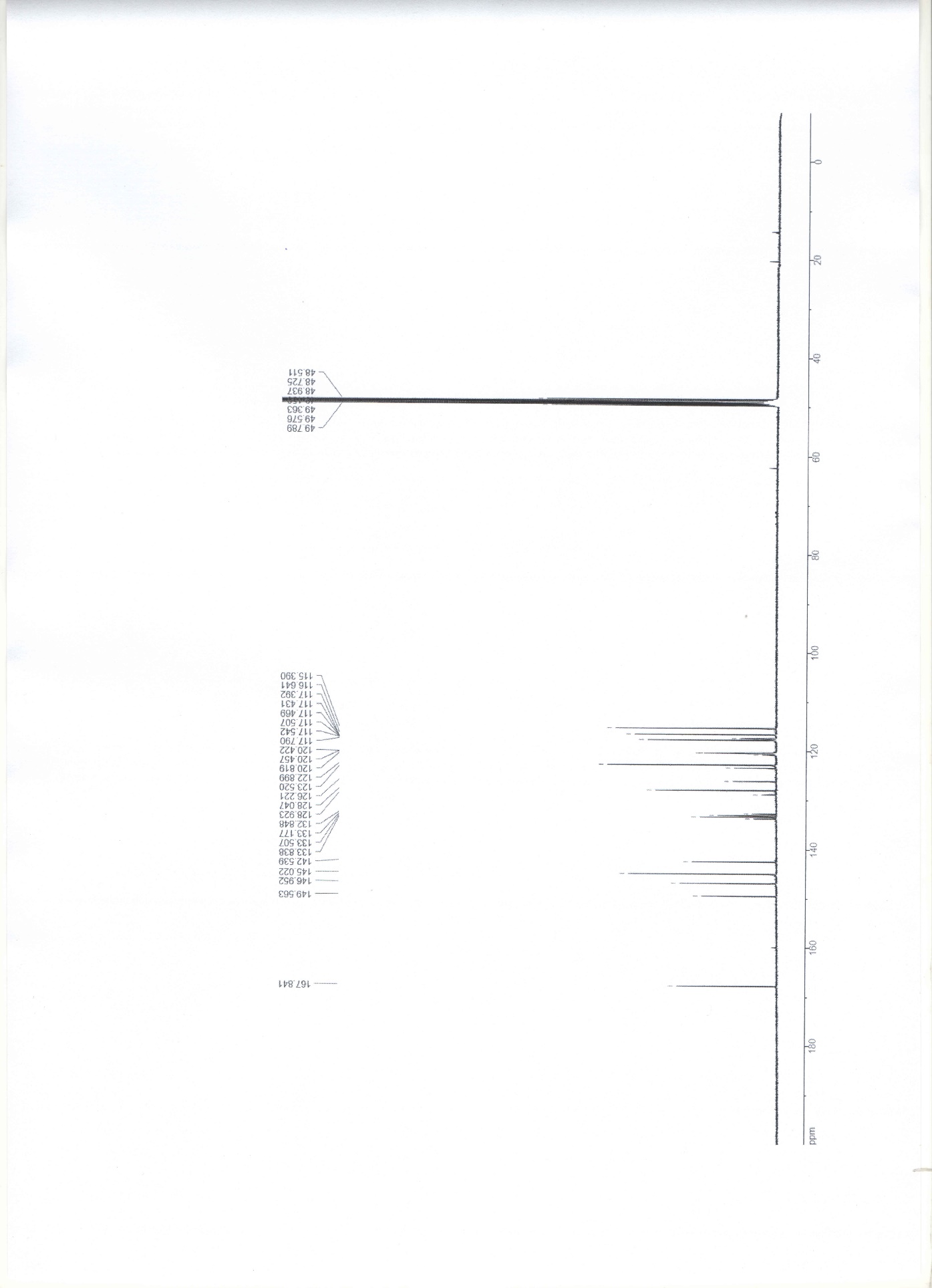


**Figure S8** ^13^C−NMR spectrum of **4** in CD_3_OD


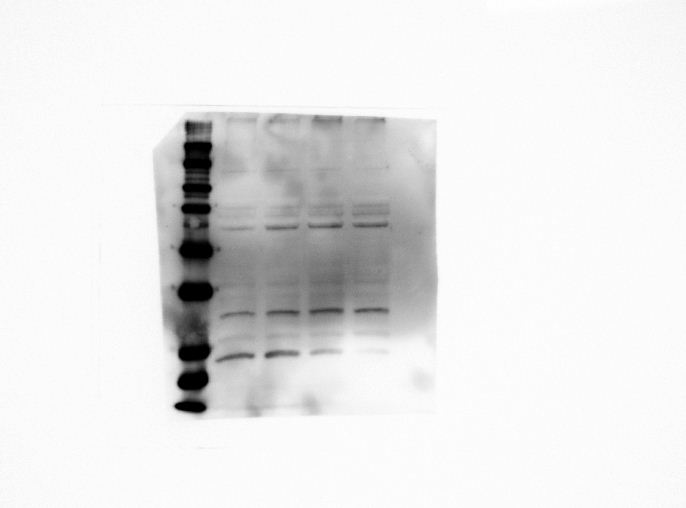

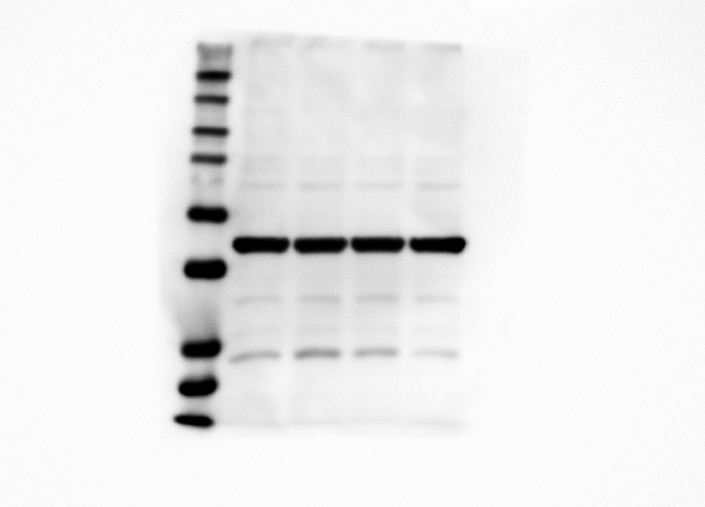


**Actin**

**SRD5A1**

**Figure S9** Original Western blot for detection of SRD5A1 protein expression at 12 h.


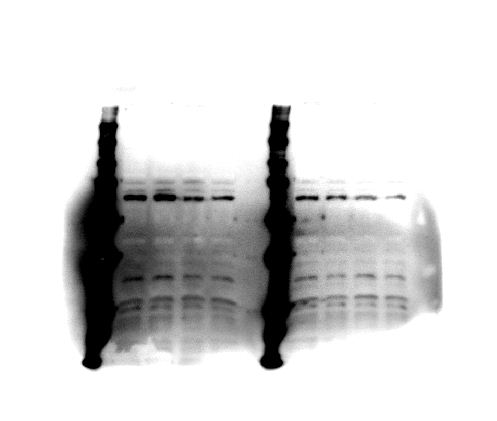

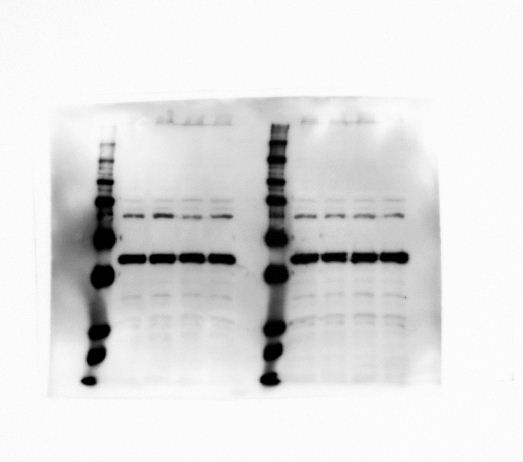


**Actin**

**SRD5A1**

**Figure S10** Original Western blot for detection of SRD5A1 protein expression at 24 h.


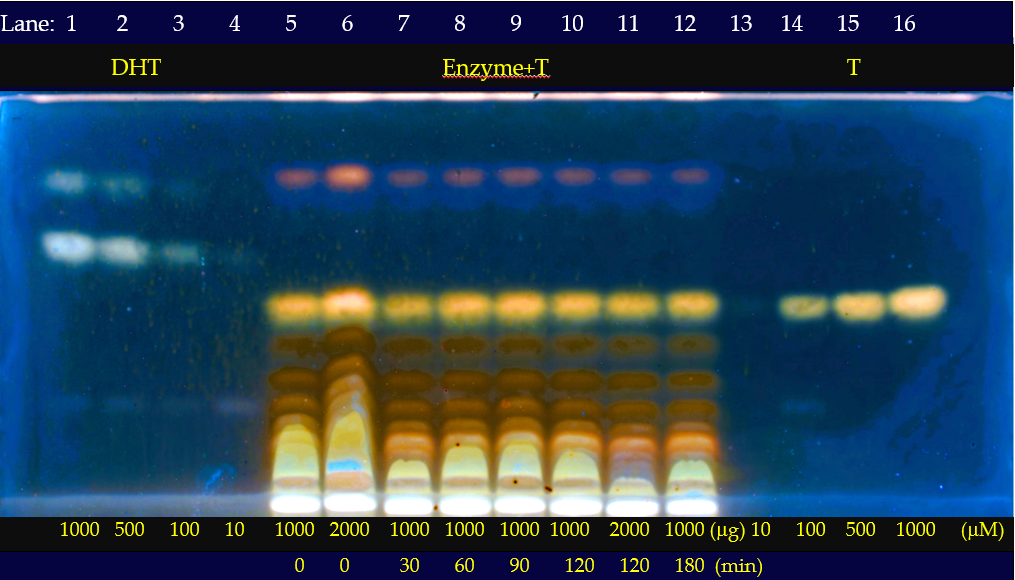


**Crude** **SRD5A1 + T**

**Figure S11** HPTLC chromatogram for the evaluation of DHT intensity by SRD5A1 enzyme-based assay using crude enzyme extracted from HaCaTs. Lanes 1−4, dihydrotestosterone (DHT) standards at 1000, 500, 100, and 10 μM. Lanes 5–12, crude SRD5A1 enzyme treated with 10 μM testosterone (T). Lanes 13−16, testosterone (T) standards at 10, 100, 500, and 1000 μM. The obtaining chromatogram was visualized by CAMAG HPTLC Software vision CATS. The HPTLC chromatogram showed no formation of DHT.


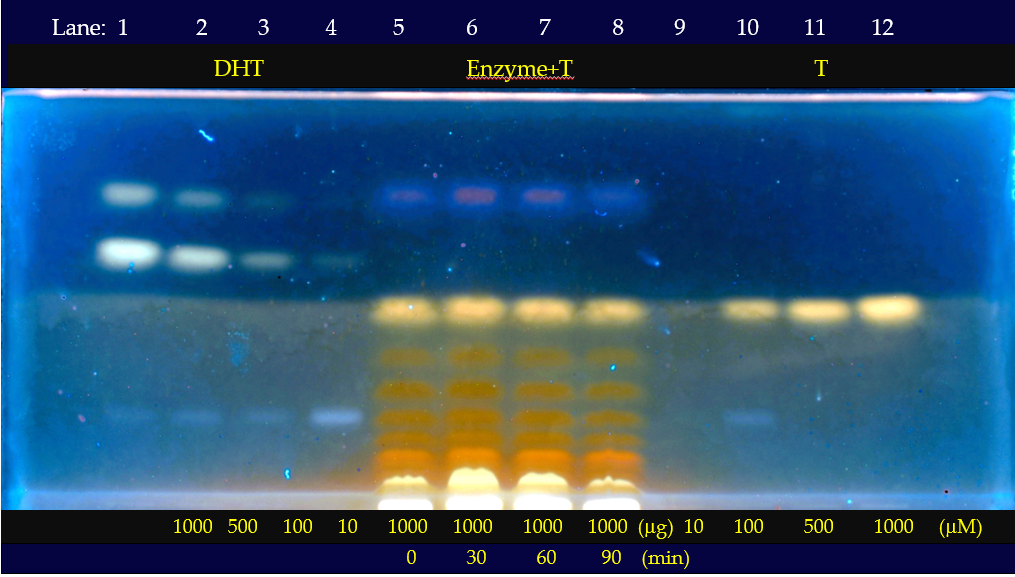


**Purified SRD5A1 + T**

**Figure S12** HPTLC chromatogram for the evaluation of DHT intensity by SRD5A1 enzyme-based assay using purified enzyme extracted from HaCaTs. Lanes 1−4, dihydrotestosterone (DHT) standards at 1000, 500, 100, and 10 μM. Lanes 5–8, purified SRD5A1 enzyme treated with 10 μM testosterone (T). Lanes 9−12, testosterone (T) standards at 10, 100, 500, and 1000 μM. The obtaining chromatogram was visualized by CAMAG HPTLC Software visionCATS. The HPTLC chromatogram showed no formation of DHT.


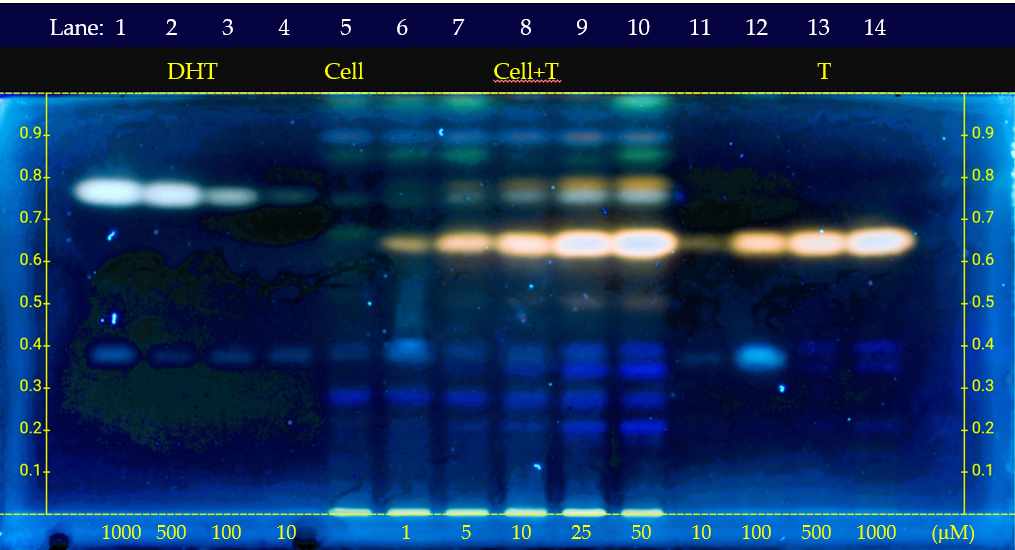


**Cell+T**

**Figure S13** HPTLC chromatogram for the HaCaT cell based kinetic study of compound **4** against SRD5A1 with various testosterone concentrations without inhibitor. Lanes 1−4, dihydrotestosterone (DHT) standards at 1000, 500, 100, and 10 μM. Lane 5, Cell refers to HaCaTs. Lanes 6−10, HaCaTs treated with testosterone at 1, 5, 10, 25 and 50 μM. Lanes 11−14, testosterone (T) standards at 10, 100, 500, and 1000 μM. The obtaining chromatogram was visualized by CAMAG HPTLC Software vision CATS.


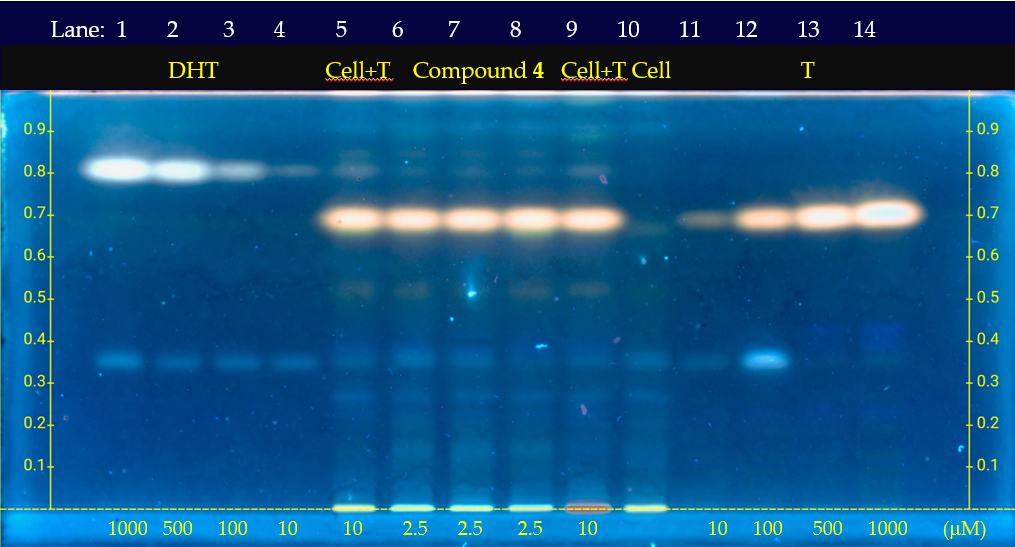


**Figure S14** HPTLC chromatogram for the HaCaT cell based kinetic study of compound **4** against SRD5A1 with compound **4** (2.5 μM) and testosterone (10 μM) at 6 h. Lanes 1−4, dihydrotestosterone (DHT) standards at 1000, 500, 100, and 10 μM. Lanes 5 and 9, HaCaTs treated with testosterone. Lanes 6−8, HaCaTs treated with compound **4**. Lane 10, Cell refers to HaCaTs. Lanes 11−14, testosterone (T) standards at 10, 100, 500, and 1000 μM. The obtaining chromatogram was visualized by CAMAG HPTLC Software vision CATS.


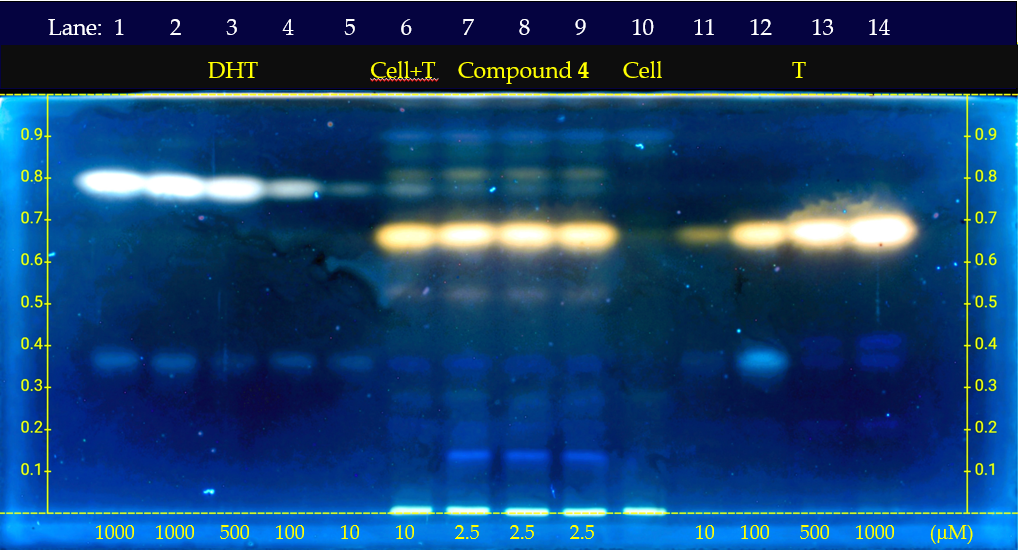


**Figure S15** HPTLC chromatogram for the HaCaT cell based kinetic study of compound **4** against SRD5A1 with compound **4** (2.5 μM) and testosterone (10 μM) at 12 h. Lanes 1−4, dihydrotestosterone (DHT) standards at 1000, 500, 100, and 10 μM. Lanes 5 and 9, HaCaTs treated with testosterone. Lanes 6−8, HaCaTs treated with compound **4**. Lane 10, Cell refers to HaCaTs. Lanes 11−14, testosterone (T) standards at 10, 100, 500, and 1000 μM. The obtaining chromatogram was visualized by CAMAG HPTLC Software vision CATS.


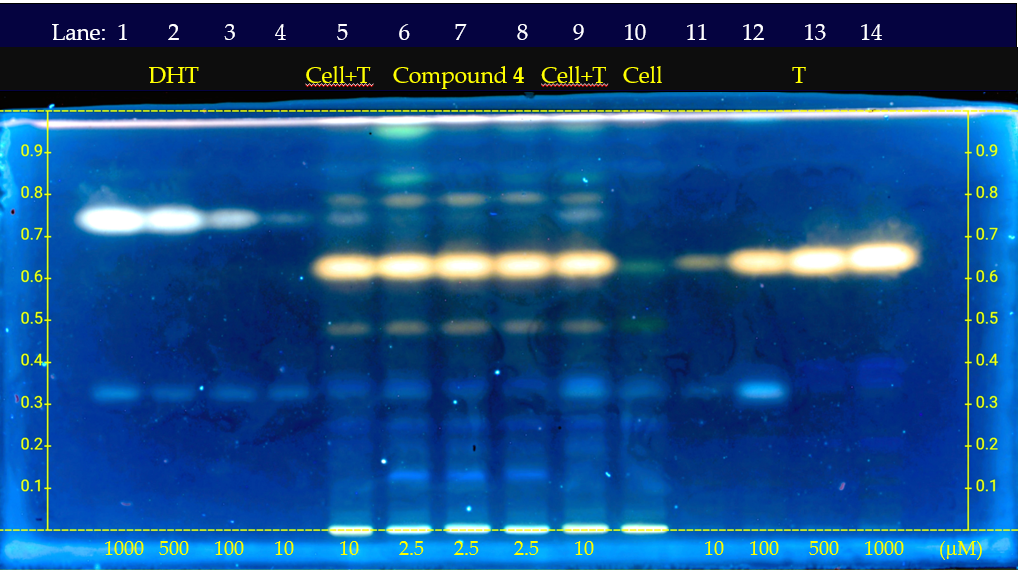


**Figure S16** HPTLC chromatogram for the HaCaT cell based kinetic study of compound **4** against SRD5A1 with compound **4** (2.5 μM) and testosterone (10 μM) at 12 h. Lanes 1−4, dihydrotestosterone (DHT) standards at 1000, 500, 100, and 10 μM. Lanes 5 and 9, HaCaTs treated with testosterone. Lanes 6−8, HaCaTs treated with compound **4**. Lane 10, Cell refers to HaCaTs. Lanes 11−14, testosterone (T) standards at 10, 100, 500, and 1000 μM. The obtaining chromatogram was visualized by CAMAG HPTLC Software vision CATS.


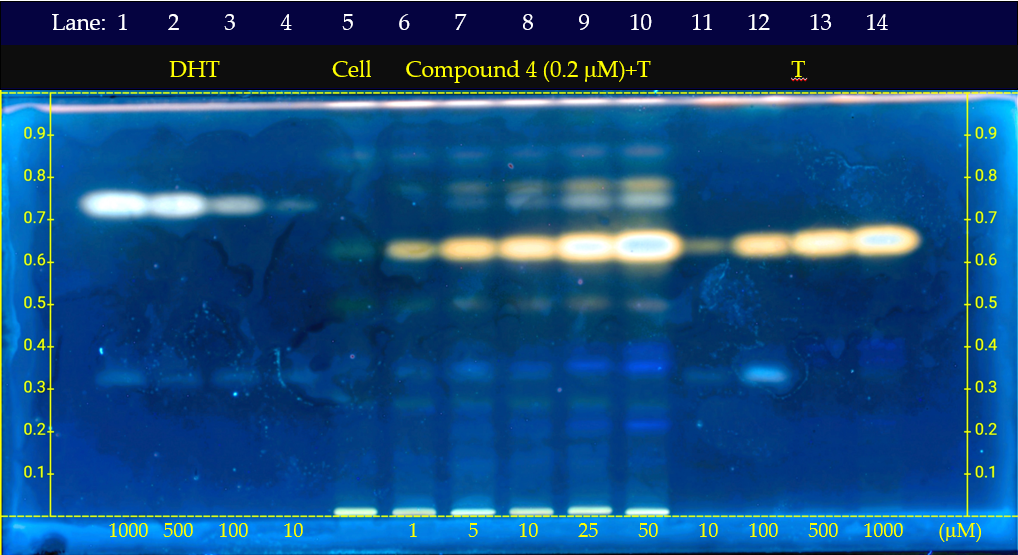


**T**

**Figure S17** HPTLC chromatogram for the HaCaT cell based kinetic study of compound **4** against SRD5A1 with various testosterone concentrations and compound **4** (0.2 μM). Lanes 1−4, dihydrotestosterone (DHT) standards at 1000, 500, 100, and 10 μM. Lane 5, Cell refers to HaCaTs. Lanes 6−10, HaCaTs treated with testosterone at 1, 5, 10, 25 and 50 μM. Lanes 11−14, testosterone (T) standards at 10, 100, 500, and 1000 μM. The obtaining chromatogram was visualized by CAMAG HPTLC Software vision CATS.


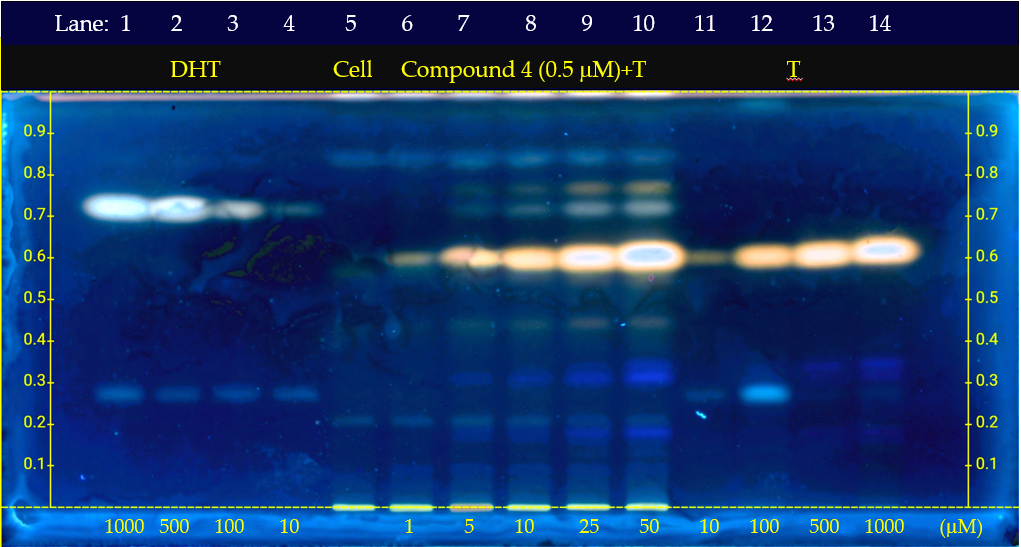


**T**

**Figure S18** HPTLC chromatogram for the HaCaT cell based kinetic study of compound **4** against SRD5A1 with various testosterone concentrations and compound **4** (0.5 μM). Lanes 1−4, dihydrotestosterone (DHT) standards at 1000, 500, 100, and 10 μM. Lane 5, Cell refers to HaCaTs. Lanes 6−10, HaCaTs treated with testosterone at 1, 5, 10, 25 and 50 μM. Lanes 11−14, testosterone (T) standards at 10, 100, 500, and 1000 μM. The obtaining chromatogram was visualized by CAMAG HPTLC Software vision CATS.


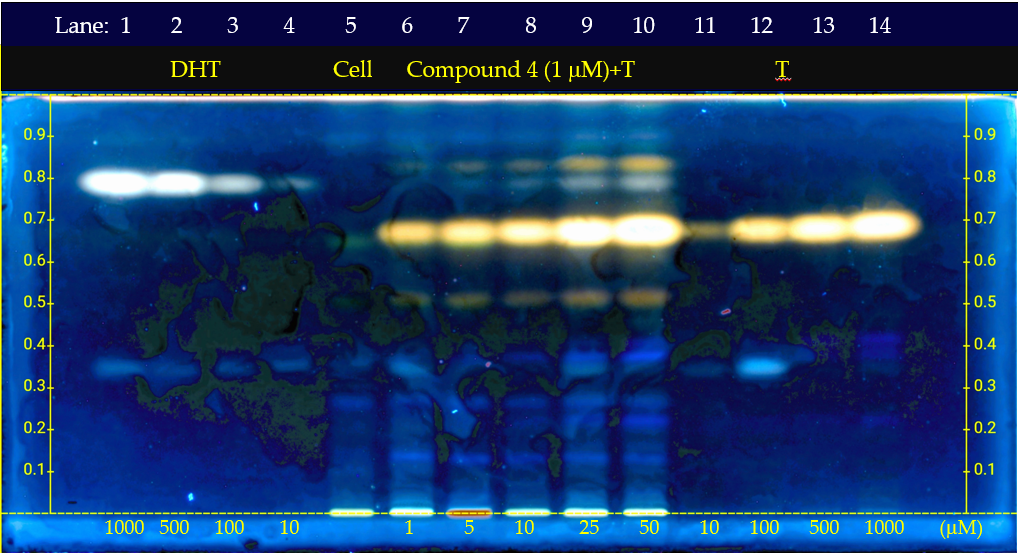


**T**

**Figure S19** HPTLC chromatogram for the HaCaT cell based kinetic study of compound **4** against SRD5A1 with various testosterone concentrations and compound **4** (1.0 μM). Lanes 1−4, dihydrotestosterone (DHT) standards at 1000, 500, 100, and 10 μM. Lane 5, Cell refers to HaCaTs. Lanes 6−10, HaCaTs treated with testosterone at 1, 5, 10, 25 and 50 μM. Lanes 11−14, testosterone (T) standards at 10, 100, 500, and 1000 μM. The obtaining chromatogram was visualized by CAMAG HPTLC Software vision CATS.


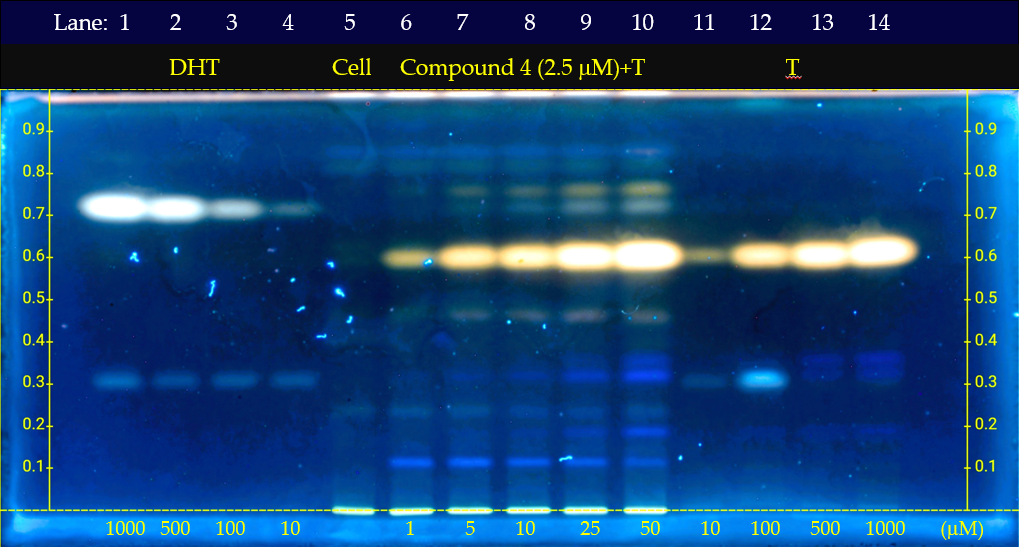


**Figure S20** HPTLC chromatogram for the HaCaT cell based kinetic study of compound **4** against SRD5A1 with various testosterone concentrations and compound **4** (2.5 μM). Lanes 1−4, dihydrotestosterone (DHT) standards at 1000, 500, 100, and 10 μM. Lane 5, Cell refers to HaCaTs. Lanes 6−10, HaCaTs treated with testosterone at 1, 5, 10, 25 and 50 μM. Lanes 11−14, testosterone (T) standards at 10, 100, 500, and 1000 μM. The obtaining chromatogram was visualized by CAMAG HPTLC Software vision CATS.


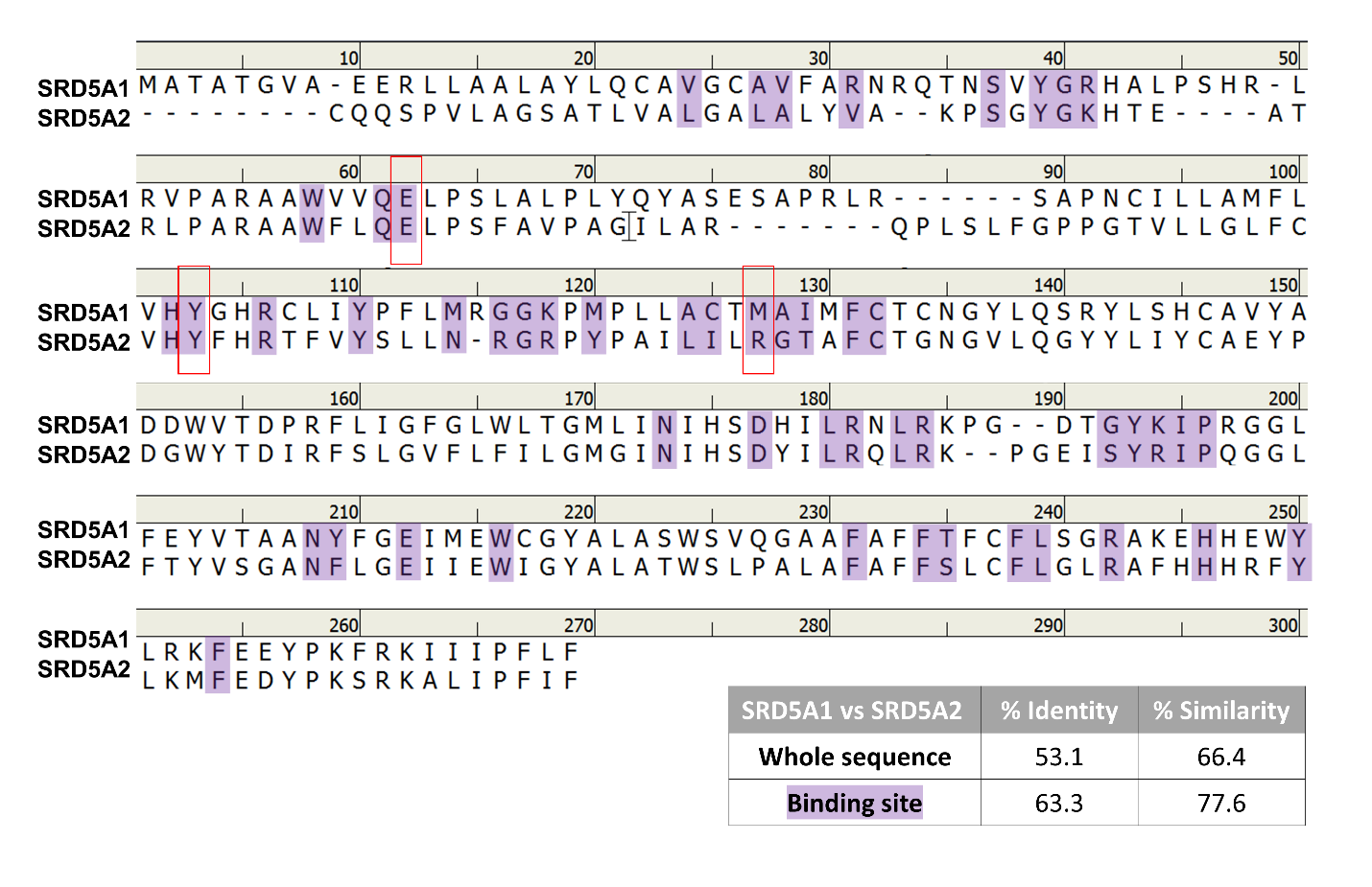


**Figure S21** Sequence alignment between human SRD5A1 and SRD5A2. The red box represents the catalytic residues of SRD5A1 (E60, Y95 and M119) and SRD5A2 (E57, Y91 and R114).

**
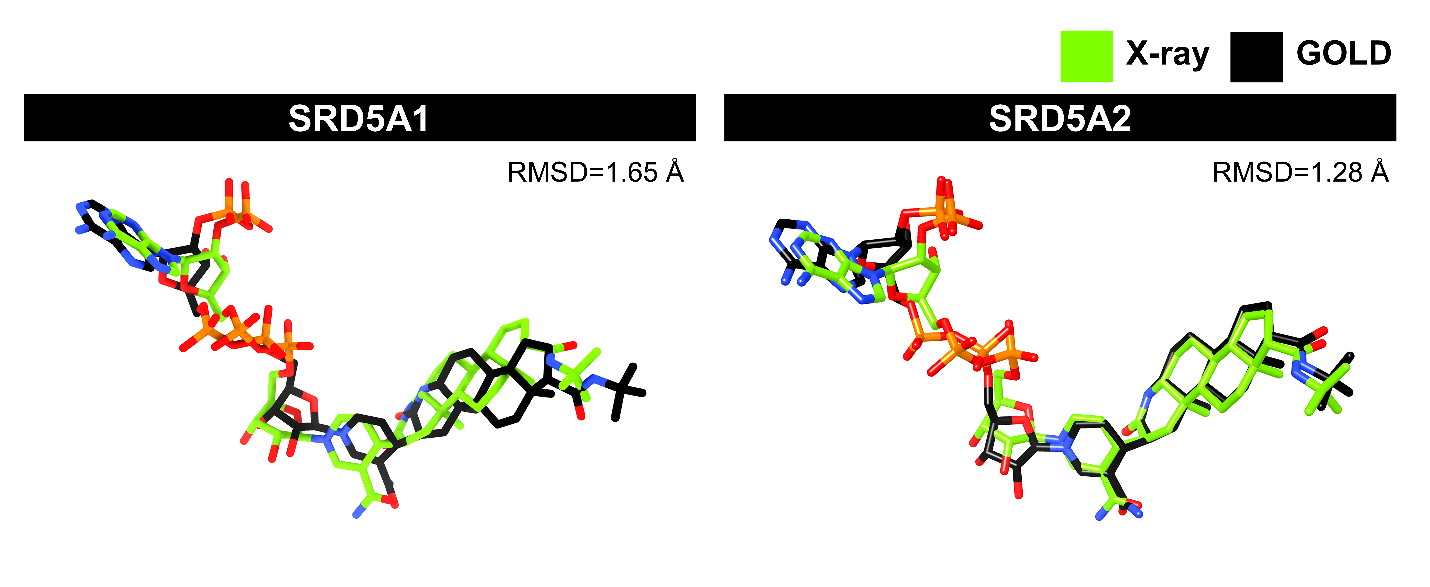
**

**Figure S22** Superimposition of NADP-dihydrofinasteride adduct against SRD5A1 and SRD5A2 the redocking pose (black) with the crystal structure of ligand (green).


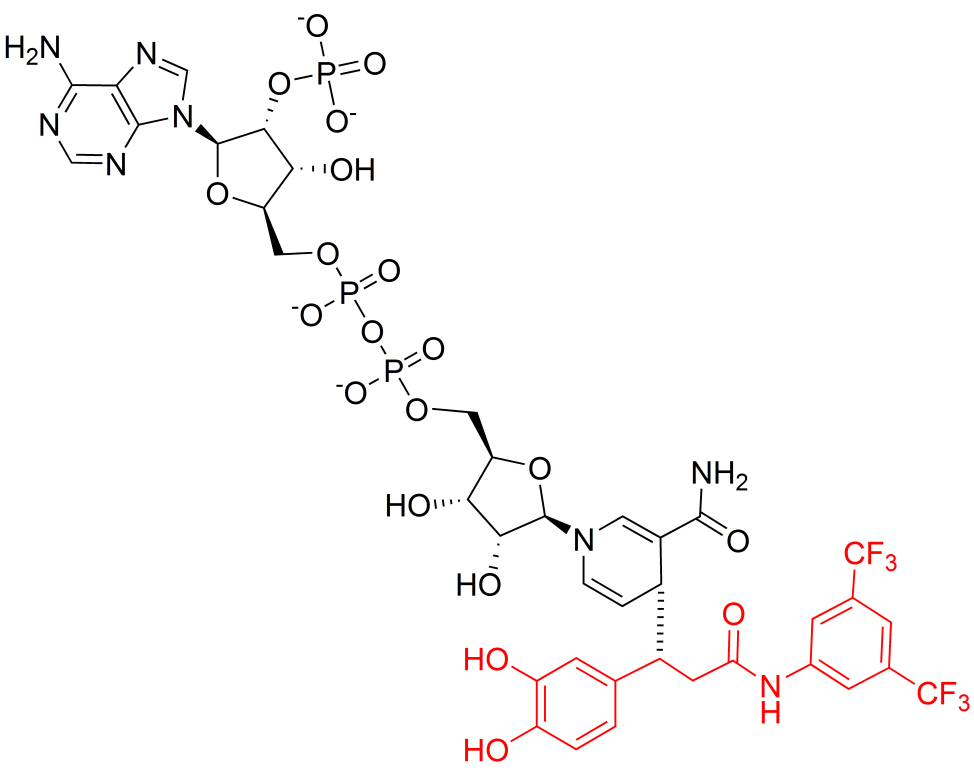


**Figure S23** 2D structure of NADP-dihydro-**4**.

**
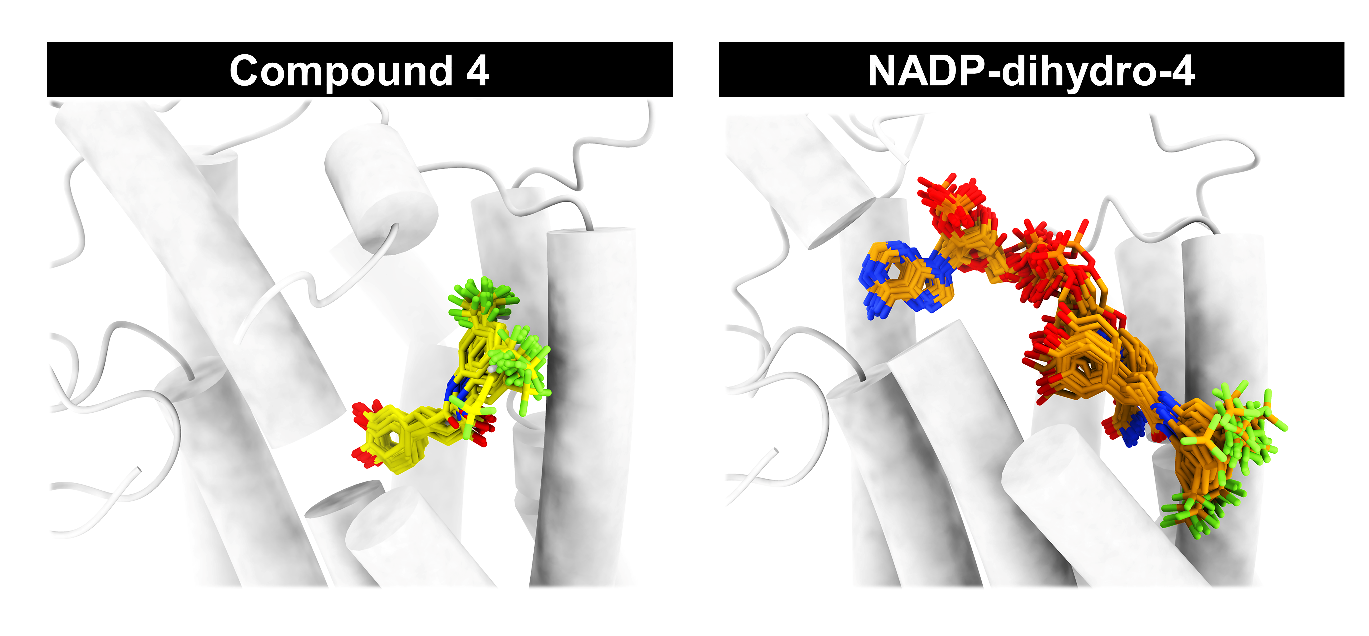
**

**Figure S24** Snapshot from the molecular dynamics (MD) trajectory derived from last 100 ns simulation (Run 1) of compound **4** and its adduct NADP-dihydro-**4** against SRD5A1.

**
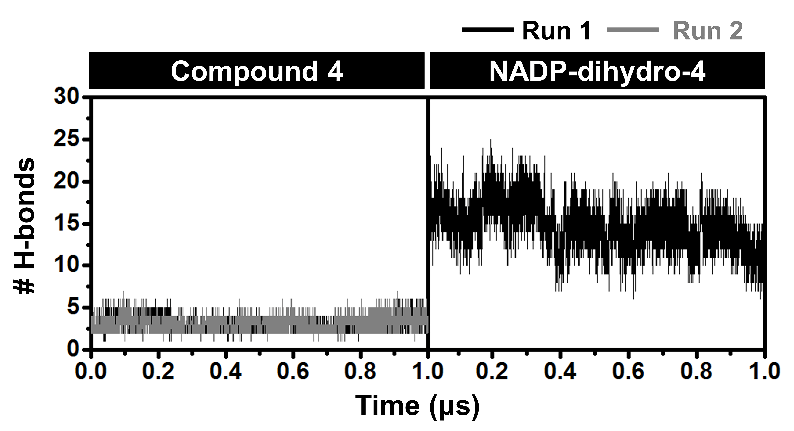
**

**Figure S25** The plots of #H-bonds of compound **4** (Runs 1 and 2) and NADP**-**dihydro**-4** (Run 1) in complex with SRD5A1 along with the 1**-**μs MD simulations. The angle between the hydrogen bond donor (HD) and hydrogen acceptor (HA) were employed as a criterion for strong hydrogen bond calculations of NADP**-**dihydro**-4** system, with the distance and angle of ≤ 3.5 Å and ≥ 120^o^, respectively.

**
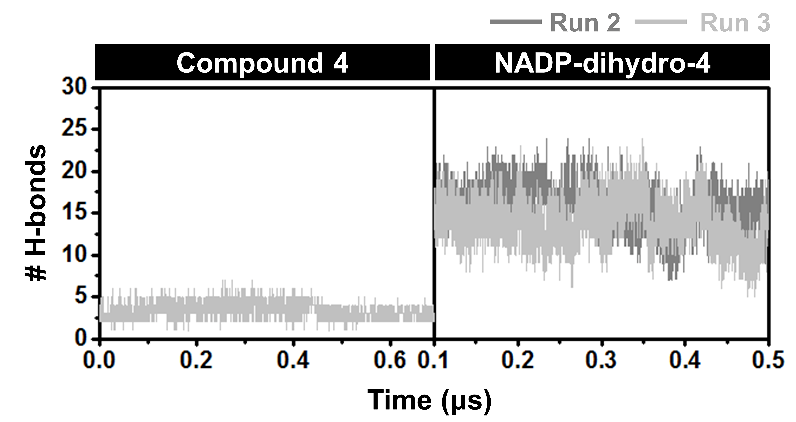
**

**Figure S26** The plots of #H-bonds of compound **4** and NADP**-**dihydro**-4** in complex with SRD5A1 along with the 0.7**-**μs (Run 3) and 0.5**-**μs (Runs 2 and 3) MD simulations, respectively. The angle between the hydrogen bond donor (HD) and hydrogen acceptor (HA) were employed as a criterion for strong hydrogen bond calculations of NADP**-**dihydro**-4** system, with the distance and angle of ≤ 3.5 Å and ≥ 120^o^, respectively.


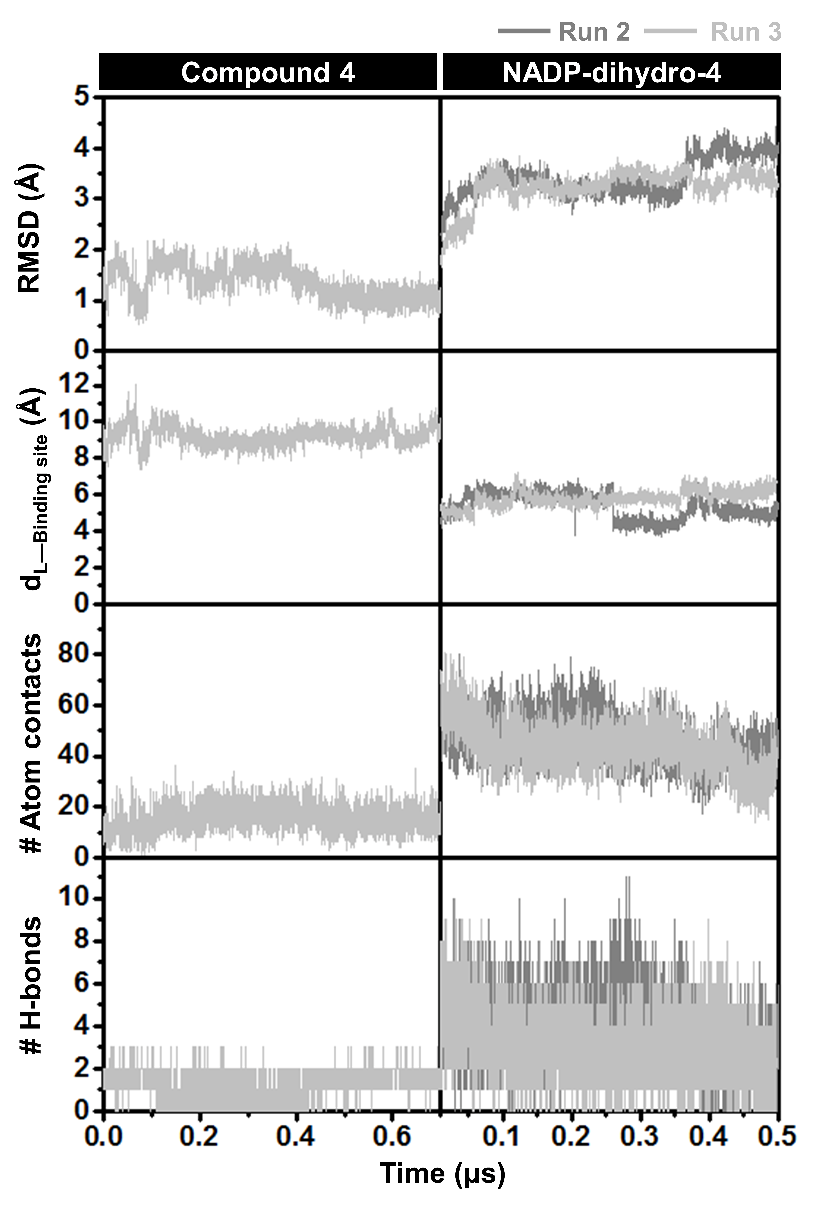


**Figure S27** The plots of the RMSD of compound **4** and NADP**-**dihydro**-4**, the distance measured between the centers of mass of compound 4 and NADP-dihydro**-**4 and binding residues (d_L-Binding site_), # Atom contacts and #H-bonds of compound **4** and NADP**-**dihydro**-4** in complex with SRD5A1 along with the 0.7**-**μs (Run 3) and 0.5**-**μs (Runs 2 and 3) MD simulations, respectively. The angle between the hydrogen bond donor (HD) and hydrogen acceptor (HA) were employed as a criterion for strong hydrogen bond calculations of NADP-dihydro**-4** system, with the distance and angle of ≤ 2.8 Å and ≥ 150^o^, respectively.

**Table S1** Dihydrotestosterone (DHT) formation from the HaCaT cell based kinetic study with various testosterone (substrate) concentrations. Cells were treated at 12 h.

| Testosterone  (µM) | % DHT intensity ± S.D. |
| --- | --- |
| 0 | 0 |
| 1 | 15.35 ± 9.10 |
| 5 | 43.55 ± 10.80 |
| 10 | 65.05 ± 13.06 |
| 25 | 103.52 ± 20.66 |
| 50 | 105.75 ± 11.30 |

**Table S2** Dihydrotestosterone (DHT) formation from the HaCaT cell based kinetic study with various testosterone (substrate) concentrations and various compound **4** concentrations.

| Compound **4** (µM)  Testosterone (µM) | % DHT intensity ± S.D. | | | |
| --- | --- | --- | --- | --- |
|  | 0.2 | 0.5 | 1.0 | 2.5 |
| 1 | 0.83 ± 0.219 | 0.70 ± 0.16 | 0.12 ± 0.01 | 0.13 ± 0.03 |
| 5 | 29.00 ± 1.829 | 44.57 ± 9.66 | 34.49 ± 3.82 | 0.31 ± 0.04 |
| 10 | 48.11 ± 5.339 | 23.04 ± 2.83 | 27.21 ± 2.98 | 30.48 ± 5.08 |
| 25 | 86.19 ± 4.08 | 71.91 ± 2.00 | 68.49 ± 0.95 | 46.20 ± 0.91 |
| 50 | 90.49 ±10.32 | 72.72 ± 12.38 | 65.61 ± 2.06 | 55.01 ± 2.40 |

Cells were treated at 12 h.

**Table S3** Lipinski’s rule of five prediction of caffeic acid, its amide derivatives and steroid 5α-reductase inhibited drugs

| **Compounds** | **Molecular Descriptors** | | | | | | |
| --- | --- | --- | --- | --- | --- | --- | --- |
|  | **Mw** | **Log P** | **No of HBA** | **No of HBD** | **RB** | **PSA** | **Lipinski’s rule of five** |
| 1 | 180.16 | 0.93 | 4 | 3 | 2 | 77.76 | Yes |
| 2 | 255.27 | 2.18 | 3 | 3 | 4 | 69.56 | Yes |
| 3 | 235.28 | 1.83 | 3 | 3 | 4 | 69.56 | Yes |
| 4 | 391.26 | 4.28 | 9 | 3 | 6 | 69.56 | Yes |
| Finasteride | 372.54 | 3.31 | 2 | 2 | 3 | 58.20 | Yes |
| Dutasteride | 528.53 | 5.71 | 8 | 2 | 6 | 58.20 | No (Mw>500, Log P >5) |

Molecular weight (Mw), Hydrogen bond acceptor (HBA), Hydrogen bond donor (HBD), rotatable bond (RB) and polar surface area (PSA).

**Supporting Video description**

**Video S1.** MD simulation of compound **4/**SRD5A1 complex derived from the last 100 ns of the Run 1 simulation.

**Video S2.** MD simulation of NADP-dihydro-**4**/SRD5A1 complex derived from the last 100 ns of the Run 1 simulation.

**Supporting File description**

**File S1.** The mdcrd file of compound **4/**SRD5A1 complex derived from the last 100 ns of the Run 1 simulation.

**File S2.** The prmtop file of compound **4/**SRD5A1 complex derived from the last 100 ns of the Run 1 simulation.

**File S3.** The mdcrd file of NADP-dihydro-**4/**SRD5A1 complex derived from the last 100 ns of the Run 1 simulation.

**File S4.** The prmtop file of NADP-dihydro-**4/**SRD5A1 complex derived from the last 100 ns of the Run 1 simulation.
