# Supplementary material for: Caffeic acid N-[3,5-bis(trifluoromethyl)phenyl] amide as a non-steroidal inhibitor for steroid 5α-reductase type 1 using a human keratinocyte cell-based assay and molecular dynamics
Source: Sci Rep. 2022 Dec 2;12:20858. doi: 10.1038/s41598-022-25335-7 (PMC9718795; doi:10.1038/s41598-022-25335-7)
Supplement: Supplementary file 8 — Supplementary Information 6. [file 41598_2022_25335_MOESM8_ESM.docx]

**Supplementary Materials:** All data generated or analyzed during this study are included in this published article and its supplementary information file: Preliminary screening of SRD5A1 inhibitory activity (**Figure S1**), Full-length and no exposure adjustment HPTLC chromatogram of caffeic acid *N*-[3,5-bis(trifluoro methyl)phenyl] amide (**4**) toward SRD5A1 inhibitory activity using a HaCaT-based assay (**Figure S2)**, ^1^H and ^13^C-NMR spectra of compounds **2**−**4** (**Figures S3**–**S8**), Original Western blot (**Figures S9** and **S10**), HPTLC chromatograms (**Figures S11**–**S20**), additional data for modeling and simulation (**Figures S21**−**27, Videos S1−S2** and **Files S1−S4**), HaCaT cell based kinetic study (**Tables S1** and **S2**) and Lipinski’s rule of five prediction of compound **1**−**5** (**Table S3**).
